# Genome sequencing analysis of *Streptomyces coelicolor* mutants that overcome the phosphate-depending vancomycin lethal effect

**DOI:** 10.1186/s12864-018-4838-z

**Published:** 2018-06-14

**Authors:** Fernando Santos-Beneit

**Affiliations:** 1 Centre for Bacterial Cell Biology, Medical School, Newcastle University, Newcastle Upon Tyne, UK; 2 Present address: Área de Microbiología, Departamento de Biología Funcional, Facultad de Medicina, Universidad de Oviedo, Oviedo, Spain

**Keywords:** Vancomycin, Teicoplanin, Glycopeptide, Phosphate, Lysozyme, SCO1213, *murT*, *gatD*

## Abstract

**Background:**

Glycopeptide antibiotics inhibit bacterial cell-wall synthesis, and are important for the treatment of infections caused by multi drug-resistant strains of enterococci, streptococci and staphylococci. The main mechanism by which bacteria resist the action of glycopeptides is by producing a modified cell-wall in which the dipeptide D-Alanine-D-Alanine is substituted by D-Alanine-D-Lactate or D-Alanine-D-Serine. Recently, it has been shown that inorganic phosphate (Pi) induces hypersensitivity to vancomycin in *Streptomyces coelicolor* (which is highly resistant to the antibiotic in low-Pi media). This finding was surprising because the bacterium possesses the entire set of genes responsible for vancomycin resistance (VR); including those coding for the histidine kinase/response regulator pair VanS/VanR that activates the system.

**Results:**

This work shows that high Pi amounts in the medium hamper the activation of the *van* promoters and consequently inhibit VR in *S. coelicolor*; i.e. the repression effect being stronger when basic or acidic forms of the nutrient are used. In addition, this work shows that lysozyme resistance is also highly regulated by the Pi concentration in the medium. At least five different mutations contribute to the overcoming of this repression effect over VR (but not over lysozyme resistance). Therefore, the interconnection of VR and lysozyme resistance mechanisms might be inexistent or complex. In particular, two kinds of mutant in which Pi control of VR has been lost (one class expresses the *van* genes in a constitutive manner; the other retains inducibility by vancomycin) have been isolated and further characterized in this study. Sequencing revealed that the first class of mutation conferred a single amino acid substitution in the second transmembrane helix of the VanS protein; whereas the other class hampered the expression or activity of a putative homolog (SCO1213) to the staphylococcal GatD protein. Complementation, phenotypic and bioinformatics analyses identified SCO1213, and its upstream gene (i.e. *murT*), as relevant genetic determinants involved with VR in *S. coelicolor*.

**Conclusion:**

The genomic approach of this study together with other genetic and phenotypic analyses has allowed the identification of the uncharacterized *murT*-*gatD Streptomyces* genes and the characterization of their involvement with the Pi control of VR in *S. coelicolor*.

**Electronic supplementary material:**

The online version of this article (10.1186/s12864-018-4838-z) contains supplementary material, which is available to authorized users.

## Background

Antimicrobial resistance (AMR) is a critical health issue today. Many important pathogens have become resistant to all available antibiotics used clinically, a problem becoming even more serious considering the lack of new antibiotics in development. Therefore, there is an urgent need to develop new antimicrobial strategies against bacteria exhibiting AMR. Vancomycin is a glycopeptide used as one of the last resort antibiotic treatments for many life-threatening bacterial infections including those caused by methicillin-resistant *Staphylococcus aureus* (MRSA); a major killer in nosocomial infections [1]. However, vancomycin resistance (VR) has spread to *S. aureus* and other important pathogens such as *Enterococcus faecium* and *Enterococcus faecalis* [2]. During the last two decades, VR *Enterococcus* (VRE) has become a major nosocomial pathogen worldwide, mainly due to their adaptability in hospital environments and limited treatment options. Numerous VR gene cluster types have been identified in enterococci; VanA and VanB being the most prevalent clusters in clinical isolates. VanA-type strains are resistant to vancomycin as well as to other glycopeptides, such as teicoplanin. On the other hand, VanB-type strains exhibit resistance to vancomycin but retain susceptibility to teicoplanin and other analogues (unless the VR gene cluster is first activated with vancomycin). Therefore, the VanA gene cluster is characterized by an inducible high level of resistance to both vancomycin and teicoplanin (the two main glycopeptide antibiotics used in the clinic) while the VanB cluster confers inducible resistance only to vancomycin [3]. Worryingly, both VanA and VanB clusters are often located in transferable and conjugative genetic elements, such as Tn*1546* and Tn*1549* transposons [4]. Consequently, dissemination of these clusters constitutes a major health risk worldwide.

In addition to pathogens, the explosion in microbial genome sequencing has also revealed the presence of Van clusters in non-pathogenic bacteria; not only in glycopeptide-producing actinomycetes such as *Amycolatopsis orientalis* and *Streptomyces toyocaensis*, but also in strains that do not produce any glycopeptide antibiotics, such as *Streptomyces coelicolor* [5, 6]. This bacterium holds a Van cluster (*vanSRJKHAX*) conferring inducible resistance to vancomycin but not to teicoplanin (similar to the phenotype shown in VanB-type VRE). Therefore, *S. coelicolor* offers a safe and convenient model system for the study of VanB-type glycopeptide resistance [5]. Previously, this specie has been widely used as a model for studies on antibiotic production in the actinomycetes; i.e. microorganisms producing over two-thirds of the clinically useful antibiotics of natural origin today and other bioactive secondary metabolites, such as antifungal compounds, antitumor agents and immuno-suppressants [7].

In *S. coelicolor*, and other bacteria, the Van cluster is controlled by a classical two-component system (VanS-VanR) that regulates the transcriptional activation of all the genes of the cluster. In the absence of vancomycin, constitutive phosphorylation of the response regulator VanR is suppressed by the phosphatase activity of VanS and as a result, there is no transcription of the Van cluster. On exposure to vancomycin (but not to teicoplanin, in the case of type B strains), VanS activity switches from phosphatase to kinase, resulting in the accumulation of phosphorylated VanR that binds to the *van* promoters and activates transcription, including that of the conserved *vanSR* and *vanHAX* core genes. *vanH* encodes a dehydrogenase that converts pyruvate to D-Lactate (D-Lac), *vanA* codes for a ligase that binds D-Alanine (D-Ala) and D-Lac amino acids and *vanX* encodes a dipeptidase that cleaves D-Ala-D-Ala dipeptides to ensure that only altered peptidoglycan precursors ending in D-Ala-D-Lac are accumulated. Overall VanHAX enzymes collectively reconstruct the bacterial peptidoglycan to terminate in D-Ala-D-Lac, in place of the canonical D-Ala-D-Ala, which is required for vancomycin binding and antibiotic action. The binding of vancomycin to D-Ala-D-Lac is reduced by approximately 1000 fold [8]. VanK is also required for VR in *Streptomyces* because this protein adds the glycine branch to cell-wall precursors ending in D-Ala-D-Lac. FemX, which is responsible for adding glycine to the normal cell-wall precursors ending in D-Ala-D-Ala, cannot perform this reaction on the modified cell-wall precursors terminating in D-Ala-D-Lac [9]. VanJ is not involved in VR but, in as yet unknown manner, is involved in teicoplanin resistance in *S. coelicolor* [10].

Transcriptional regulation of VR is not as simple as a couple of *van* promoters activated under the presence of vancomycin by a single transcriptional regulator (i.e. *vanR*). Recently, it has been demonstrated that VR in *S. coelicolor* and *Streptomyces lividans* is highly influenced by the inorganic phosphate (Pi) concentration of the culture medium. VR was essentially blocked in these bacteria with Pi concentrations above 0.25% [11, 12]. Interestingly, addition of Pi to the medium significantly increased vancomycin sensitivity in both *S. coelicolor* wild-type and Δ*phoP* mutant strains. Therefore, this regulatory effect is not driven (in principle) by the main Pi-responding transcriptional regulator characterized in bacteria (i.e. PhoP) and alternative players may be involved. The aim of this work is to study *S. coelicolor* mutants that overcome the Pi-depending vancomycin lethal effect so as to bring new insights into the interrelated role of Pi and VR. The work also aims to check if the Pi control observed in the VR mechanism is extended to other cell-wall acting compounds, such as lysozyme, in *S. coelicolor*.

## Results

### Pi enhances the activity of vancomycin and lysozyme against *S. coelicolor*

It has previously been shown that combination of vancomycin with increasing concentrations of dipotassium hydrogen orthophosphate (K_2_HPO_4_) from 0 to 1% augments the zone of growth inhibition against *S. coelicolor* M145 in a disc diffusion assay [12]. The main Pi source in that work was K_2_HPO_4,_ which has a pH of ~ 8.4 when 1 g is dissolved in 100 mL of water (i.e. 1%). To investigate whether the inhibitory effect on VR is specific for PO4^3−^ or, for example, a pH effect, *S. coelicolor* M145 was grown in Difco Nutrient Agar (DifcoNA) with and without 50 μg per mL of vancomycin in the presence of various Pi salts, as follows: a) No Pi salt (pH~ 6.8), b) 1% K_2_HPO_4_ (pH~ 8.2), c) 1% KH_2_PO_4_ (pH~ 5.4), d) 1% K_2_HPO_4_ + KH_2_PO_4_ (pH~ 6.8), e) 1% Na_2_HPO_4_ (pH~ 8.2), f) 1% NaH_2_PO_4_ (pH~ 5.4), g) 1% Na_2_HPO_4_ + NaH_2_PO_4_ (pH~ 6.8). The strain grew in a similar manner in all media when no vancomycin was added, indicating that these salts are non-toxic at a 1% concentration. The strain also grew normally in the presence of vancomycin when no Pi salt was added. However, no growth occurred when any of the basic or acid sodium or potassium forms of Pi were added to the vancomycin supplemented media; although the growth was only partially blocked with the addition of the neutral pH salts. Therefore, this result discards a specific role for potassium or sodium in the negative effect on VR and, on the other hand, points to an important role for monobasic or dibasic Pi in the observed inhibitory effect.

To further test the negative effect of K_2_HPO_4_ (i.e. the Pi salt used in the previous report by Santos-Beneit and Martín; 12) in VR, the *S. coelicolor* M145 cells were subjected to a Pi gradient experiment in DifcoNA supplemented with 50 μg per mL of vancomycin. For this experiment, the spores were diluted to obtain (when adding 100 μL of the spore suspension to the medium) around 100 colonies growing per control plate. The same amount of spores (i.e. 100 μL) was streaked in the vancomycin supplemented cultures containing an increasing gradient of Pi from 0 to 1% (with an increasing unit of 0.1%). The result showed that no surviving colonies were observed when a Pi amount of 0.2% or higher was added to the medium. In the condition of 0.1% Pi the number of growing colonies was reduced to > 50% (respect to the control condition of no Pi addition). In summary, no extremely high amounts of Pi are required to significantly decrease VR in *S. coelicolor*.

To check if this negative effect of Pi on VR is also observed with other cell-wall acting compounds, such as lysozyme, the resistance pattern of *S. coelicolor* was tested in the presence of an increasing concentration of lysozyme (up to 500 μg per mL) and with or without supplementation of 1% K_2_HPO_4_. As shown in Table 1 *S. coelicolor* M145 cultures without Pi supplementation grew with all lysozyme concentrations tested, while with Pi supplementation growth was only observed on a lysozyme concentration up to 10 μg per mL. Therefore, it seems that lysozyme resistance is also regulated by K_2_HPO_4_ in *S. coelicolor*.

**Table 1:** Pi regulation of vancomycin and lysozyme resistance. The concentration of the compounds is shown as μg per mL. (Pi = K_2_HPO_4_)

DifcoNA								
Lys	Van	WT	W1	W2	L1	L2	L4	L5
–	–	++++	++++	++++	++++	++++	++++	++++
10	–	++++	++++	++++	++++	++++	++	++++
100	–	++++	++++	++++	++	++	+	++
500	–	++++	++++	++++	+	–	–	+
–	50	++++	++++	++++	++++	++++	++++	++++
10	50	++++	++++	++++	++	+	–	++
100	50	++	++	++	+	–	–	+
500	50	++	++	++	–	–	–	–
DifcoNA + 1%Pi								
Lys	Van	WT	W1	W2	L1	L2	L4	L5
–	–	++++	++++	++++	++++	++++	++++	++++
10	–	++++	++++	++++	++	–	–	++
100	–	–	–	–	–	–	–	–
500	–	–	–	–	–	–	–	–
–	50	–	–	–	++++	++++	++++	++++
10	50	–	–	–	–	–	–	–
100	50	–	–	–	–	–	–	–
500	50	–	–	–	–	–	–	–

### Construction of a vancomycin inducible *vanSR*-*vanJ*_p_-*neo* fusion reporter system

**Fig. 1:**
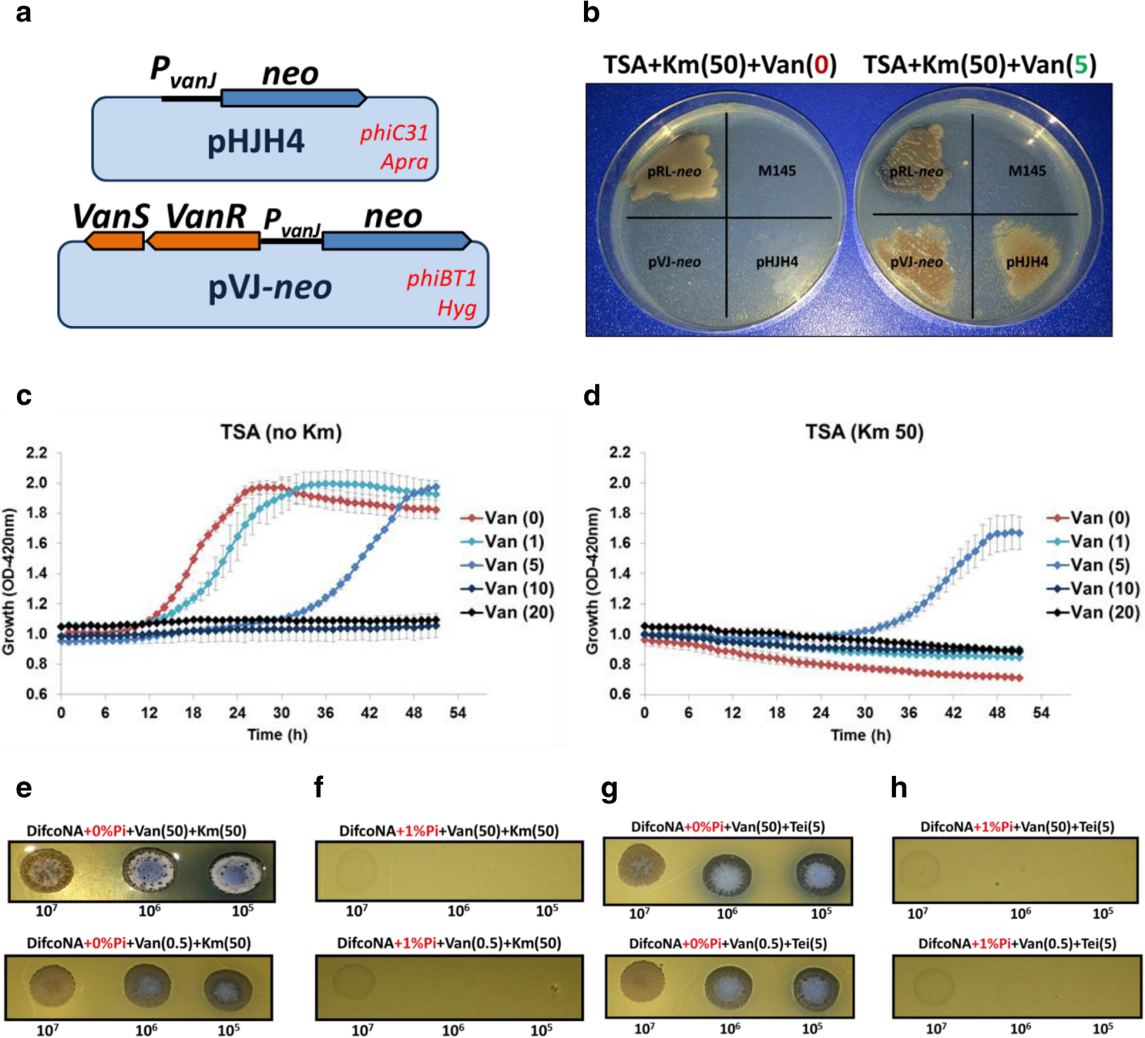
**a** Schematic representation of the pVJ-*neo* and pHJH4 reporter systems. **b** Checking of pVJ-*neo* and pHJH4 reporter systems in TSA plates. The same amount of spores (10^6^) of *S. coelicolor* strains containing pVJ-*neo*, pHJH4, pRL-*neo* and no vector were inoculated in TSA kanamycin cultures (50 μg per mL) with or without 5 μg per mL of vancomycin. Plates were analysed after 4 days of growth at 30 °C. **c**-**d** Induction of pVJ-*neo* using 96-well microplates. Different concentrations of vancomycin (0, 1, 5, 10 and 20 μg per mL) were added to TSA containing either no kanamycin (**c**) or 50 μg per mL of kanamycin (**d**). In each well was added 10^6^ spores of *S. coelicolor* W1. Plates were then incubated at 30 °C during 52 h. Growth was determined by optical density (420 nm) using a BMG Fluostar Optima fluorometer. (**e**-**f**) *S. coelicolor* W1 cells were grown during 6 days in Difco nutrient agar containing kanamycin (50 μg per mL) and vancomycin (50 μg per mL, upper panels) / (0.5 μg per mL, lower panels) and with (**f**) or without 1% K_2_HPO_4_ (**e**) addition. Three different amount of spores (10^5^, 10^6^ and 10^7^) were inoculated in each case. **g-h** Same as e-f but with the addition of teicoplanin (5 μg per mL) instead of kanamycin

To examine whether the negative effect of Pi on VR is exerted at the promoter level a vancomycin reporter system was constructed and named pVJ-*neo* (Fig. 1a). The system carries a 2 kbp fragment of the *S. coelicolor van* cluster containing the *vanSR* genes and the *vanJ* promoter (*vanJ*_*p*_). The aminoglycoside phosphotransferase gene (*neo*), which confers resistance to both neomycin and kanamycin, was transcriptionally fused to *vanJ*_*p*_ in pVJ-*neo*. In parallel, a similar reporter construct was also exploited (pHJH4; see Fig. 1a). This vector was previously shown to confer resistance to kanamycin in *S. coelicolor* in the presence of vancomycin (and other *van* inducers) but not in their absence [13]. The two systems differ in that the pVJ-*neo* system carries second copies of the *vanS*-*vanR* gene system. This feature might be beneficial in mimicking the natural organization of the divergent *vanSR*-*vanJ* promoter region. Also, having two copies of the regulatory system should limit the recovery of mutations in these immediate regulators in favour of other classes of mutation. The vector pVJ-*neo* was introduced in *S. coelicolor* M145 giving the strain *S. coelicolor* W1. On the other hand, the vector pHJH4 was introduced in *S. coelicolor* M145 giving *S. coelicolor* W2.

Functionality of the two reporter systems was first tested on kanamycin (50 μg per mL) supplemented TSA (for the composition of this medium see Methods). This medium has the advantage of having a relative high Pi concentration (0.25% K_2_HPO_4_) with a neutral pH; therefore, the effect of a high or low pH is avoided. To have an estimation of the amount of vancomycin needed to induce resistance in this medium the MIC_50_ value was calculated (the MIC_50_ represents the minimum inhibitory concentration value at which 50% of the isolates in a test population are inhibited). The MIC_50_ value obtained in TSA for both *S. coelicolor* W1 and W2 strains was around 5 μg per mL of vancomycin. Then, to check the functionality (and plausible differences of inducibility) of the two reporter systems described above, *S. coelicolor* W1 and W2 cells (and a control strain expressing the *neo* gene in a constitutive manner; i.e. pRL-*neo*) were grown in TSA supplemented with 50 μg per mL of kanamycin (with either no vancomycin or 5 μg per mL of antibiotic). The results (Fig. 1b) showed that both pHJH4 and pVJ-*neo* vectors supported induction of the *neo* gene in a vancomycin dependent manner (the strains grew in the plates with vancomycin but not in the plates without the inducer). Therefore, both pHJH4 and pVJ-*neo* vectors provide a way to readily monitor induction of the *vanJ* promoter via resistance to kanamycin.

The pVJ-*neo* system developed in this work was further checked by growing the *S. coelicolor* W1 cells in a polystyrene sterile 96-well plate with TSA medium and different concentrations of vancomycin and/or kanamycin. Plates were incubated at 30 °C for 52 h and growth was determined by optical density using an automatic microplate reader. Growth of the strain is shown in Fig. 1c-d (as a mean value of 8 replicates for each condition). The amount of vancomycin that was required to confer resistance to kanamycin ranged from > 1 to < 10 μg per mL of antibiotic. Actually, induction of *vanJ* promoter was only obtained when a vancomycin amount of 5 μg per mL was added to the medium (i.e. others vancomycin concentrations between 1 and 10 μg per mL were not tested).

As a second approach to check the pVJ-*neo* system the strength of *vanJ* induction was analysed using DifcoNA (in which VR is not compromised in such low vancomycin concentrations as with TSA). Different concentrations of vancomycin (0.5 or 50 μg per mL) and K_2_HPO_4_ (0 or 1%) were added to the medium. As shown in Fig. 1e, the W1 cells were able to induce the *vanJ* promoter (i.e. conferring kanamycin resistance) with either 0.5 or 50 μg per mL of vancomycin when no Pi was added to the medium. However, the induction of *vanJ* promoter by vancomycin (either with 0.5 μg or 50 μg per mL) was completely lost in the medium with 1% K_2_HPO_4_ (Fig. 1f). It is important to note that an amount 0.5 μg per mL of vancomycin does not kill the cells in DifcoNA+ 1% K_2_HPO_4_ when no kanamycin is added (which indicates that the lack of growth in the condition of kanamycin addition is due to an insufficient *vanJ* promoter activation and no because an inhibitory effect of vancomycin at this concentration).

To determine whether induction of teicoplanin resistance by vancomycin (VanB-type strains induce teicoplanin resistance in a vancomycin-dependent manner) is also under a negative control by Pi in *S. coelicolor* the same approach was employed but, instead of adding kanamycin to the cultures, teicoplanin was added. As previously, the W1 cells were able to induce teicoplanin resistance with 0.5 or 50 μg per mL of vancomycin in DifcoNA but not in DifcoNA+ 1% K_2_HPO_4_ (Fig. 1g-h). In summary, the results indicate that the induction range of the *van* promoters in *S. coelicolor* is quite diminished in high Pi concentrations.

### Selecting mutants inducing *vanJ*_p_ at high pi concentrations

**Fig. 2:**
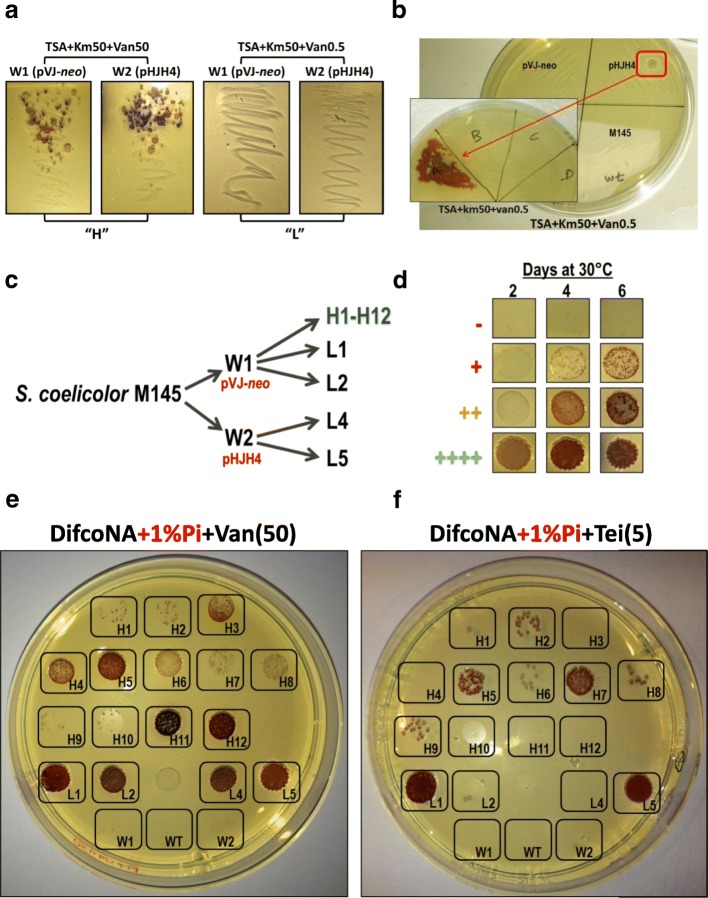
**a** Selection of mutant strains inducing *vanJ*_p_ in high Pi concentrations. 10^8^ spores of *S. coelicolor* W1 and W2 strains were streaked in TSA containing kanamycin (50 μg per mL) and vancomycin (50 or 0.5 μg per mL). Plates were grown at 30 °C until mutant colonies could be observed. **b** Isolation of “L” mutants after increasing the amount of the inoculum (i.e. 10^9^ spores) and repeating the experiment several times. In the figure is shown the isolation of the L5 mutant as an example. **c** Scheme of the “H” and “L” mutants isolated and analysed in this study. **d** Scheme representing the four main growth patterns observed for the strains in the growth conditions of the study, which are summarized with the next symbols (−, +, ++ and ++++). **e**-**f** Analysis of the growth of the mutant strains incubated at 30 °C during 4 days in: (**e**) DifcoNA with 1% K_2_HPO_4_ and 50 μg per mL of vancomycin or in (**f**) DifcoNA with 1% K_2_HPO_4_ and 5 μg per mL of teicoplanin

A series of mutants were selected to gain insights into the regulatory mechanisms conferring Pi control on the activity of the *van* promoters. These mutants were selected in TSA using two different inhibitory growth conditions: a) kanamycin + 0.5 μg per mL of vancomycin (i.e. ten times lower than the MIC_50_); b) kanamycin + 50 μg per mL of vancomycin (i.e. ten times higher than the MIC_50_). As shown in Fig. 2a, the number of putative mutant strains growing with the high vancomycin concentration was much higher than with the lower concentration (indeed no colonies at all grew with the low vancomycin amount in a first experiment). By increasing the amount of the inoculum, and by several repetitions of the experiment, four mutant strains were isolated in the low vancomycin condition (see Fig. 2b-c). The isolation of mutants with the capability to grow in the higher vancomycin concentration was performed by picking 12 random colonies from those that were able to grow in the experiment shown in Fig. 2a (i.e. using only the panel of the W1 strain; see Fig. 2c). All the mutants selected (termed “H” or “L” for high or low vancomycin concentrations, respectively) passed two rounds of selection in their respective antibiotic concentrations in order to select for stable genotypes/phenotypes. After selecting the mutants, the MIC value of vancomycin (shown as μg per mL of antibiotic) in TSA was calculated for all the strains: a) W1, W2 and M145 ≤ 10. b) “H” mutants > 50 and < 100. c) “L” mutants > 100. These results indicate that all the “L” strains are more resistant to vancomycin than the “H” strains in the selective TSA medium.

As a first approach, to check the Pi control on VR in the selected mutant strains, the growth of the parental (W1, W2 and M145) and mutant strains was analysed in both TSA and DifcoNA (with or without 1% K_2_HPO_4_) using different concentrations of vancomycin (and with or without kanamycin or teicoplanin to monitoring the inducibility of the *van* promoters). All the “L” strains and half of the “H” strains were able to grow (in a lower or higher extent) in DifcoNA+ 1% K_2_HPO_4_ with 50 μg per mL of vancomycin (see Fig. 2e); contrary to the parental strains. Seven (H5, H11, H12, L1, L2, L4 and L5) out of these strains showed a fully growth profile (++++) according to the pattern shown in Fig. 2d, which indicates that differences in the regulatory mechanisms might exist between all the selected strains.

**Fig. 3:**
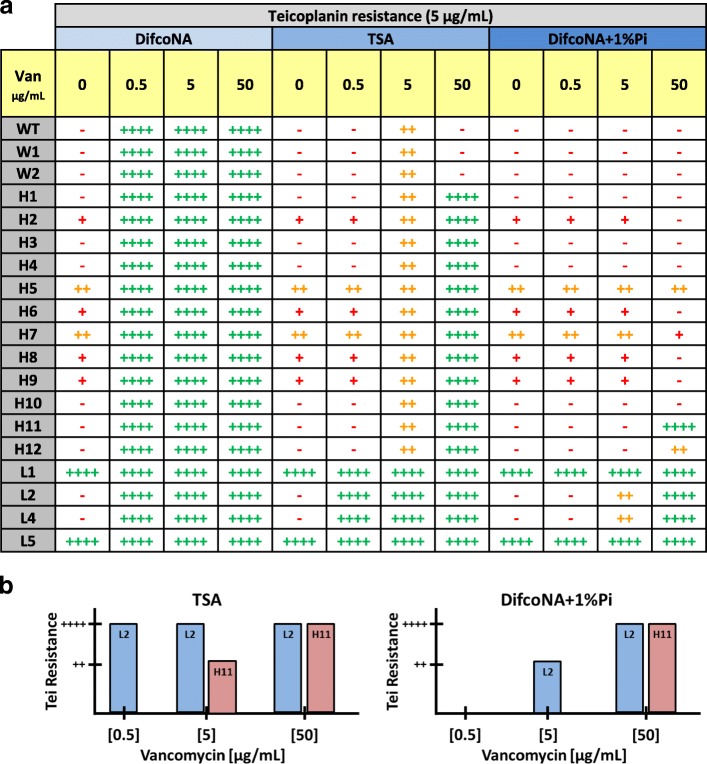
**a** Growth patterns obtained for the mutant and parental strains in DifcoNA supplemented with 5 μg per mL of teicoplanin (with or without 1% K_2_HPO_4_ addition) and TSA supplemented with 5 μg per mL of teicoplanin. In all cases different concentrations of vancomycin were combined. A similar result was observed with the addition of kanamycin instead of teicoplanin and for this reason the data is not shown. **b** Schematic representation of the induction of teicoplanin resistance in the “L2” and “H11” strains with TSA and DifcoNA+ 1% K_2_HPO_4_

To further distinguish between mutants that maintained the inducibility by vancomycin from those showing a constitutive *van* induction phenotype, the cells were grown with teicoplanin and without vancomycin (teicoplanin itself does not induce the *van* genes). The results showed mutants L1 and L5, in contrast to the rest of the strains, with the ability to fully grow in teicoplanin without the inducer (see Fig. 2f). A similar result was obtained when kanamycin, instead of teicoplanin, was used (data not shown). This result suggests that L1 and L5 mutants are probably inducing the *van* genes in a constitutive manner. When testing the inducibility of teicoplanin (or kanamycin) resistance with low (0.5) or high (50) vancomycin concentrations only the “L” mutants were able to grow in all media combinations (except in DifcoNA+ 1% K_2_HPO_4_ + Van0.5 in which only L1 and L5 grew; see Fig. 3a). Curiously, only one “H” mutant strain (H11) was able to induce teicoplanin (and kanamycin) resistance at the highest extent (++++) in DifcoNA+ 1% K_2_HPO_4_ + Van50; none were able to do it with 0.5 or 5 μg per mL of the antibiotic (see Fig. 3a-b). These results indicate that the “L” mutants are not only more resistant to vancomycin than the “H” strains, but also have a much wider induction range of the *van* promoters.

As a second approach, to detect additional differences between the mutants, the cells (including the parental strains) were subjected to a combination of Pi, lysozyme and vancomycin in DifcoNA. Lysozyme resistance of all the “L” mutants was decreased in relation with the parental strains in all growth conditions; especially in L2 and L4 (see Table 1). This indirect correlation between VR and lysozyme resistance was not conserved in all the “H” strains analysed. Actually, at least two “H” mutants (H11 and H12) showed an increased resistance to lysozyme in comparison to the parental strain in both low and high Pi conditions (data not shown). Therefore, the interconnection of these regulatory mechanisms might be inexistent or complex.

### Identification of gene polymorphisms associated with increased VR in high Pi media

Since the acquired VR phenotypes of “L” and “H” strains remained stable during sub-cultures it was expected to find changes in the genome sequences of these strains. As a first approach to identify the genomic alterations responsible for the VR phenotypes Sanger sequencing of the *vanSR* genes and all the *van* promoters of the cluster was performed. Curiously, this first analysis showed that the strains L1 and L5 had acquired the same single nucleotide polymorphism (SNP) during the selection procedure. This SNP resulted in the substitution of glycine to aspartic acid in the amino acid 77 of the VanS protein; which was not observed for the rest of strains (see Table 2). Two different PCR reactions for each of the L1 and L5 strains were performed in order to exclude a mistake of the polymerase reaction rather than a real mutation in the chromosome (see Additional file 1: Figures S1-S2). A different pair of primers (hybridizing in the pVJ-*neo* vector backbone) allowed to specifically sequencing the *vanSR* genes of the pVJ-*neo* vector (contained in L1 but not in L5). No mutational changes were detected in *vanSR* or the *vanJ* promoter of pVJ-*neo* in L1 (see Table 2). Thus, only the chromosomal pair (from the two set of *vanSR* genes present in L1) was mutated.

**Table 2:** Sanger and Illumina sequencing analyses of the “H” and “L” mutant strains. The table shows the mutations identified by both Illumina and Sanger sequencing analyses in each of the mutant strains

	Mutation in chromosomal *van genes*	Mutation in *vanSR* of pVJ	Mutation in other loci
W1	No	No	No
W2	No	–	No
H1	No	No	No
H2	No	No	?
H3	No	No	?
H4	No	No	No
H5	No	No	SCO4474 _Thr477- Lys477_
H6	No	No	?
H7	No	No	?
H8	No	No	?
H9	No	No	?
H10	No	No	?
H11	No	No	SCO3167 _(5nt-insertion)_
H12	No	No	No
L1	VanS _Gly77-Asp77_	No	No
L2	No	No	SCO1212 _Val120-Gly120_
L4	No	–	SCO1212 _(GTG→GTT)_
L5	VanS _Gly77-Asp77_	–	No

As a second approach to detect genomic alterations in the mutant strains Illumina whole genome sequence analysis of W1, W2, L2, L4, H1, H4, H5, H11 and H12 was performed (i.e. the rest of the strains were not selected for the analysis). The L5 strain was also included in the analysis as a control. The SNPs, insertions and deletions (InDels) of each individual strain (including W1 and W2) were identified relative to the reference strain *S. coelicolor* M145. By subtraction relative to the respective parental strain, sequencing differences were identified in the genome sequences of each of the mutant strains (i.e. L2 versus W1 and L4 versus W2). Only two specific mutations were detected in the selected “H” strains in relation to its W1 parental strain. One of these two mutations was found in SCO4474 of H5. This gene codes for a putative cytochrome c biogenesis ResB-like protein. The mutation (a SNP) resulted in the substitution of threonine to lysine in the amino acid 477 of the protein (see Table 2 and Additional file 1: Figure S3). The other mutation was detected in the SCO3167 gene of H11; coding for a putative *tetR*-family transcriptional regulator. The mutation produced a 5-nucleotide insertion in the 3´ region of the SCO3167 ORF (i.e. disrupting the translation of the full-length version of the gene). Sanger sequencing using as control the W1 parental strain confirmed both mutations (see Additional file 1: Figure S3-S4). The study of these two genes is not included in this manuscript and it may form the basis of future work.

Interestingly, L2 and L4 acquired during the selection procedure a different SNP but in the same gene (SCO1213). The SNP of L2 resulted in the substitution of valine to glycine in the amino acid 120 of the protein, while the SNP of L4 resulted in the change of the putative translation start triplet of SCO1213 from GTG to GTT (i.e. which might lack the translation of the protein at this triplet). Both mutations were confirmed by Sanger sequencing using as control the respective W1 and W2 parental strains (see Additional file 1: Figures S5-S7). No additional SNPs or InDels were detected for L2 or L4 in comparison with W1 and W2, respectively. In strong support of the Illumina data, the *vanS* mutation detected in L5 with the Sanger analysis was also detected by the whole genome sequence analysis (see Additional file 1: Figure S1). Similar to the L2 and L4 strains no additional variations were detected in L5 with the Illumina analysis.

### The identified SNP in *vanS* results in a constitutive activation of the van system

**Fig. 4:**
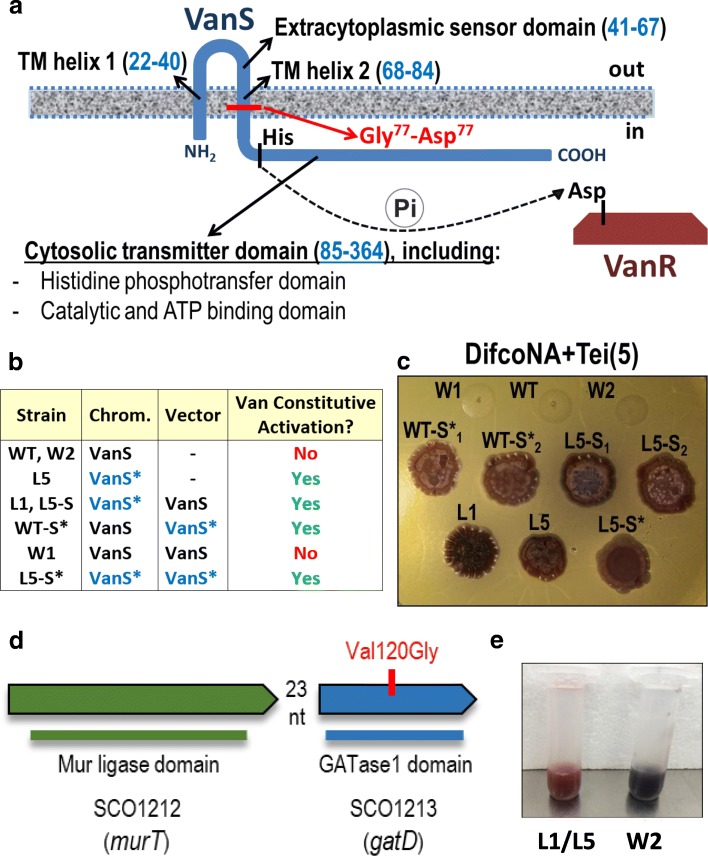
**a** Schematic representation of the localization of the mutation identified in L1 and L5 strains according to a predicted structure of the *S. coelicolor* VanS protein by Hutchings and co-workers [18]. **b** Table summarising the results of L1, L5, WT and complemented strains according to their capability to grow in teicoplanin containing plates without the presence of the inducer (i.e. vancomycin). **c** Plate showing the growth of the developed *vanS* strains in Difco nutrient agar with 5 μg per mL of teicoplanin (and no vancomycin) after 6 days of incubation at 30 °C. **d** Genetic organization of *murT* (green) *gatD* (blue) in *S. coelicolor*. The localization of the mutation of L2 in SCO1213 is shown with a vertical line. In the image is also shown the conserved domains detected for the proteins according to the NCBI’s conserved domain database [28]. **e** Red overproduction phenotype in the spore stocks of L1 and L5 in comparison to the rest of strains. In the picture, the spore stock of L5 is shown at the left and the spore stock of W2 at the right

The mutation in VanS substitutes a glycine residue by an aspartic acid residue in the second transmembrane segment of the protein (see Fig. 4a and Additional file 1: Figure S8). To check the effect of this mutation on VR it was made a series of complemented strains combining mutated or not mutated genes in single copy or in parallel with the wild type gene (see Fig. 4b). Important differences in the patterns of *van* induction were observed between the strains containing at least one mutated *vanS* gene and the strains with only the wild type copy of the gene. All the strains containing at least one mutated *vanS* gene (in parallel or not with the wild type gene) grew in the presence of kanamycin and/or teicoplanin without the addition of vancomycin (see Fig. 4c). This indicates that the mutation is dominant and produces a constitutive activation of the *van* promoters.

### SCO1213 is involved in VR

The second mutation implicated in VR of the “L” strains occurred in gene SCO1213 (see Fig. 4d), which codes for a homolog of the characterized GatD protein of *S. aureus* (i.e. both containing a Type 1 glutamine amidotransferase central domain). The *gatD* product, along with the product of its upstream gene (*murT*), amidates the α-carboxylic group of the iso-D-glutamate residue at position 2 of the peptide chain of the peptidoglycan [14, 15]. The *murT*-*gatD* genetic block is conserved in many bacteria, including *S. coelicolor*. SCO1212 and SCO1213 share 28% identity / 45% similarity and 32% identity / 48% similarity with the *S. aureus* sequences of MurT and GatD, respectively. The mutation identified in L2 substitutes the amino acid 120 located within the putative catalytic region of SCO1213 (Additional file 1: Figure S9) and, according to a Psipred [16] inference, interferes with the structure of a 7-amino acid beta strand (Additional file 1: Figure S10). On the other hand, the mutation identified in L4 disrupts the putative start codon triplet of SCO1213 (i.e. avoiding translation from this triplet). Therefore, both mutations seem to hamper the function of the protein, the first affecting its catalytic activity and the second affecting its expression. To verify the role of the mutated SCO1213 gene in the phenotypes of L2 and L4, the strains were complemented with plasmids pCOM-1213-L2 and pCOM-1213-L4, respectively. These vectors contained a proper antibiotic selection marker to be used in the respective strain (apramycin in L2 and hygromycin in L4), the intact copy of SCO1213 and the upstream gene (i.e. SCO1212) with its own promoter (the intergenic region of SCO1212–13 has 23 nt; i.e. this segment is too short to comprise a specific promoter for SCO1213). To exclude a role of SCO1212 (i.e. *murT*) in the complemented strains the control vectors contained the same inserts as the complementation vectors with the exception of the SCO1213 ORF (see Methods section). The results with the complemented strains are shown in Additional file 2: Table S1. The wild type vancomycin and lysozyme resistance phenotypes were almost fully restored in all the complemented strains relative to the control strains; i.e. pointing to a specific role of SCO1213 (i.e. GatD) in the L2 and L4 phenotypes.

### Overexpression of *murT* or *gatD* does not affect VR and lysozyme resistance

According to previous transcriptomic analyses neither SCO1212 (*murT*) nor SCO1213 (*gatD*) responded to the addition of vancomycin to the medium [17]. The transcription of both genes was maintained invariant before and after this alteration (in contrast to the *van* genes that were significantly activated by vancomycin). To gain further information about the role of *murT* and *gatD* on VR both genes were overexpressed with either a *van* inducible promoter or a constitutive promoter (in sum to the wild type copy pair). To this aim both genes were individually coupled to *vanJ* promoter in pVJ and pVJc vectors (giving pVJ-1212/pVJ-1213 and pVJc-1212/pVJc-1213, respectively). The four vectors were introduced in *S. coelicolor* M145 (i.e. containing also the wild type copies) and assayed in DifcoNA with different combinations of Pi, lysozyme, vancomycin and teicoplanin. No differences were observed in any of the strains in relation to the control strains in all conditions tested (Additional file 3: Table S2). This result indicates that exclusively the depletion of these genes, and no the overexpression, produces a significant change in the VR and lysozyme resistance profiles of the bacterium.

### Analysis of growth of the mutant strains using a microfluidic device

Interestingly, it has been noted that in most media tested the size of colonies was smaller in L2 and L4 in comparison with the rest of the strains (data not shown). A decrease in the size of the colonies was also described in another high VR mutant strain (SCO2594::Tn*5062*) that, in addition, was affected in cell-wall synthesis [17]. Furthermore, a reduced growth was also reported in *S. aureus gatD* depleted strains [14]. To get further insights on the effect of *vanS* and *gatD* mutations on the growth of *S. coelicolor* L1, L2, L4 and L5 strains a time-lapse microfluidic experiment using the commercial ONIX device was performed. In all cases TSB (the liquid form of TSA) was constantly pumped into the microfluidic device with no vancomycin supplementation (to avoid an effect of the antibiotic on the growth of the bacterium). Growth of the spores was imaged by phase-contrast time-lapse microscopy during 20 h (i.e. the images were acquired every 10 min). The images showed an aberrant growth in L2 and L4 in comparison with W1, W2, L1 and L5 (see Additional files 4, 5, 6, 7, 8 and 9: Videos S1-S6). In summary, tip elongation and growth rates seem to be diminished in L2 and L4, which points to an effect of *gatD* disruption, not only on VR, but also in the growth of the bacterium.

## Discussion

In this work the complexities of VR regulation have been investigated in a model organism that belongs to the group of bacteria that are the likely primordial source of the Van clusters [6]. The strong Pi regulation on VR reported in *S. coelicolor* makes this organism a robust and reliable model to test this phenomenon of an interconnected antibiotic resistance and nutrient regulation mechanisms. A comparative genome sequence analysis approach, based on specifically selected strains, has allowed the identification of several genetic factors (i.e. *vanS* and *gatD*) contributing to VR. These genetic factors, and also the Pi concentration in the medium, have been shown to be important mediators not only for VR but also for lysozyme resistance in *S. coelicolor*. In all the strains analysed, vancomycin acted in synergy with lysozyme to cause bacterial lysis. All the strains were less resistant to lysozyme when vancomycin was present in the medium, and/or vice versa. This is not strange since both compounds target the cell-wall and, therefore, a sum lethal effect is expected. The negative effect of Pi over both VR and lysozyme resistance suggests common mechanisms of regulation; however, mutations that were positive for increasing VR were normally negative for lysozyme resistance (see Table 1). This indirect correlation between VR and lysozyme resistance was not conserved in all the high VR strains analysed. Actually, at least two “H” mutants showed an increased resistance to lysozyme in comparison to the parental strain in both low and high Pi conditions (data not shown). Therefore, the interconnection of these regulatory mechanisms might be inexistent or complex.

There are many examples in the scientific literature of mutations in genes coding for a two-component system that produce a constitutive activation of the members that the own system positively regulates. However, it was unexpected to isolate two mutant strains, each from a different parental strain, with exactly the same SNP. This was the case with L1 and L5 mutants. These mutants were shown to have a point mutation producing the same amino acid substitution in the VanS protein. The mutation substitutes a glycine residue by an aspartic acid residue in the second transmembrane segment of the protein. Glycine is a small, hydrophobic and non-charged amino acid while aspartic acid is a negatively charged and hydrophilic amino acid. Aspartic acid residues also create putative phosphorylation sites. Therefore, a significant change in the 3D structure of the protein and/or in the function of the protein is expected with this mutation. In a previous work, Hutchings and co-workers [18] identified several mutations in *vanS* producing a constitutive activation of the *S. coelicolor* Van cluster. According to the authors these mutations had something in common; all were loss-of-function mutations. In this study, by a series of complementation experiments, it has been shown that the mutation of L1 and L5 in *vanS* is dominant; producing a constitutive activation of the *van* promoters even in the presence of the wild type gene. Additional phenotypic differences were evident in the strains carrying at least one *vanS* mutated gene; especially in terms of undecylprodigiosin production and pigmentation of the spores (see Fig. 4e).

Increased resistance to vancomycin (i.e. conserving the inducibility by the antibiotic) was acquired in two mutant strains (L2 and L4) by two different SNPs; but in the same gene (SCO1213). Other high vancomycin resistant isolates, such as the “H” mutants, were evidenced in this study. Some of these “H” strains showed a constitutive activation phenotype (as L1 and L5) and others were able to induce the *van* genes in a vancomycin dependent manner (as L2 and L4). However, in contrast with L2 and L4, none of the inducible “H” mutants (including the most resistant H11 strain) were able to induce teicoplanin resistance (or kanamycin) in TSA when just as few as 0.5 μg per mL of vancomycin was added to the medium. Something similar occurred in DifcoNA+ 1% K_2_HPO_4_; but in all cases with a ten times higher magnitude (i.e. 50 μg per mL of vancomycin was required to induce teicoplanin resistance -or kanamycin- in H11, while just 5 μg per mL were sufficient in L2 and L4; see Fig. 3a-b). These results are consistent with a scenario in which the more Pi is added to the medium the more vancomycin is needed for inducing *van* gene expression. Actually at least five times lower amounts of vancomycin were needed to induce fully teicoplanin resistance (or kanamycin) in L2 and L4 when no Pi was added to DifcoNA in comparison to the “H” mutant strains (0.1 vs 0.5 μg per mL, respectively). Therefore, by a yet unknown reason, L2 and L4 (both mutants in the SCO1213 gene) are rare exceptions among the rest of mutants analysed and can induce *van* gene expression with a lower vancomycin concentration independently of the Pi concentration.

The *S. aureus* GatD protein (i.e. the SCO1213 homolog) together with MurT (i.e. the SCO1212 homolog) are responsible for the conversion of a glutamic acid residue to a glutamine residue in the peptidoglycan of the bacterium [14, 15]. The *murT*-*gatD* genes form a syntenic block that is widespread among bacteria (see Additional file 1: Figure S11), including the actinomycetes (such as the vancomycin producer *A. orientalis* or the glycopeptide A47934 producer *S. toyocaensis*) and other Gram-positives like for example *Mycobacterium tuberculosis*, *Streptococcus pneumonia* or *Clostridium perfringens*. GatD and MurT appear to form a physically stable bi-enzyme complex and to contain all domain functions required for amidation of glutamic acid in the lipid-linked peptidoglycan precursor Lipid II [14, 15]. MurT, which exhibits a typical Mur ligase central domain including the ATP binding motif, may be responsible for the recognition of the reaction substrates (i.e. Lipid II and ATP), while GatD seems to provide the catalytic subunit involved in the transfer of the amino group from free glutamine to the peptidoglycan precursor (see Fig. 4d). Amidation of peptidoglycan glutamic acid residues is common to many bacterial species and has been shown to be required for efficient peptidoglycan cross-linking in different bacteria, such as *S. aureus* and *S. pneumonia* [14, 19]. Inhibition of amidation by depletion of *murT*-*gatD* genes caused reduced growth rate, reduced resistance to beta-lactam antibiotics and increased sensitivity to lysozyme in *S. aureus* [14]*.* In *S. pneumonia* a mutation in *spr1443* (*murT*) was shown to confer resistance to pneumophages [20]. Due to the high distribution of *murT*-*gatD* genes among bacteria, additional physiological roles for the amidation of peptidoglycan cannot be discarded. Actually, in this work an uncharacterized effect involving a mutation on *gatD* (i.e. SCO1213) has been reported. Two different *S. coelicolor gatD* mutants (L2 and L4) have been shown to be more resistant to vancomycin than the parental strains, especially in high Pi conditions. In addition, the two mutant strains showed increased sensitivity to lysozyme and reduced growth rate (similar to the phenotype described for a *S. aureus murT*-*gatD* depleted strain). Overall, the strong impact of *gatD* mutations on the growth rate of distinct bacteria suggests that an amidated peptidoglycan may provide better substrates for proteins that catalyze peptidoglycan biosynthesis [14]. Moreover, amidation of the peptidoglycan seems to increase the binding affinity of several glycopeptides (such as oritavancin and chloroeremomycin) to the amidated versions of both Lipid II and its depsipeptide counterpart (i.e. Lipid II-D-Lac) in comparison to the respective non-amidated variants [21]. However, in contrast to the observations with oritavancin and chloroeremomycin, enhanced binding affinity to amidated lipid II was not observed with vancomycin in vitro [21]. Amidation of the glutamic acid residues (which implies a reduction of the number of cell wall carboxylate groups and a less negatively charged surface) could also indirectly impact on the incorporation of large amounts of negatively charged Pi groups to the cell-wall (i.e. in the form of teichoic acids or other Pi-rich cell-wall polymers) and as a result to affect the Pi metabolism regulation of the bacterium. Whether MurT-GatD (SCO1212–13) activity, together with the effect of large amounts of Pi, actually contributes to vancomycin susceptibility in *S. coelicolor* through an increasing of the antibiotic affinity for the amidated version of the peptidoglycan precursor or through an interaction with the Pi metabolism (or through an alternative mechanism) is still unknown. Therefore, further work will be required to determine the role of MurT-GatD in VR and/or Pi regulation in *S. coelicolor*. Furthermore, it will be of great interest to check whether a vancomycin-resistant *S. aureus* (VRSA) strain (and/or VRE strains) conserves the Pi control over the VR mechanism and, in that case, if a depleted mutant in *murT*-*gatD* overcomes such negative regulation. Understanding the mechanisms for tackling the Pi-dependent VR repression effect might provide insights for the development of new strategies against antibiotic-resistant bacteria via lowering antibiotic use.

## Conclusion

The results of this study show that high Pi amounts in the medium hamper the activation of the *van* promoters and consequently inhibit VR in *S. coelicolor*; i.e. the repression effect being stronger when basic or acidic forms of the nutrient are used. It is yet unknown if the regulatory effect of the nutrient interacts or not with the lysozyme resistance mechanism that, in the same manner than VR, is highly regulated by the Pi concentration of the culture. Multiple factors, including those responsible for constitutive activation of the Van system (such as the *vanS* allele of L1/L5) and those yet unknown depending on the SCO1213 (GatD) protein, contribute for an overtaking of this Pi-dependent VR obstacle. Other genetic determinants, such as the previously described SCO2594 gene [17] and those already identified in some of the “H” mutant strains, are probably also involved with the tackle of the vancomycin lethal effect in high Pi concentrations. However, none of the mutants analysed in this study abolished the Pi control over the lysozyme resistance mechanism of the bacterium. Therefore, the interconnection of VR and lysozyme resistance mechanisms might be inexistent or complex.

## Methods

### Bacterial strains, plasmids and growth conditions

The *Escherichia coli* and *S. coelicolor* species used in this work are listed in Table 3. All the bacterial strains have been acquired from the Centre for Bacterial Cell Biology (CBCB) collection. In Additional file 10: Table S3 is shown all the primers used in this work. *E. coli* strains were cultured and transformed using standard procedures. The *vanSR*-*J* fragment for construction of pVJ was amplified by PCR using the primers FSB29 (sense) and FSB30 (antisense). The sequences for the restriction enzymes KpnI and HindIII-NdeI were introduced in FSB29 and FSB30, respectively. These primers amplified a 2074-bp fragment encompassing the *S. coelicolor vanS*-*vanR* genes and the divergent *vanR*-*vanJ* promoter. Then, a KpnI–HindIII digested fragment was cloned into pMS82, yielding pVJ (8.3 kbp). The promoter-less *neo* gene was amplified by PCR using the primers FSB48 (NdeI) and FSB49 (HindIII); a further 822 bp NdeI–HindIII digested fragment was cloned into pVJ, yielding pVJ-*neo* (9.1 kbp). The same strategy was used for the construction of pVJc and the pVJc-*neo* derivative. For pVJc the same FSB29 and FSB30 primers than for pVJ were used, but instead of using total DNA from the wild type strain it was used total DNA from the L5 mutant strain. For pRL-*neo* the *S. coelicolor rpsL* promoter was amplified by PCR using FSB88 (KpnI) and FSB89 (NdeI) primers and cloned in pVJ-*neo* digested with KpnI-NdeI (i.e. the Van fragment is substituted by the *rpsL* promoter) giving pRL-*neo* (7.6 kbp). For construction of pVJ-1212/pVJc-1212 and pVJ-1213/pVJc-1213, the SCO1212 and SCO1213 genes were amplified using primers FSB201-FSB202 and FSB203-FSB204, respectively, and introduced in either pVJ or pVJc after NdeI/HindIII digestion. For the complementation of L2 and L4 mutants a 2285 bp fragment encompassing not only SCO1213 but also SCO1212 and its promoter region from + 1 to + 102 (positions from the ATG translation start triplet of SCO1212) was amplified by PCR. For L2 complementation the BamHI (FSB184-L2; forward primer) and EcoRV (FSB185-L2; reverse primer) restriction sites were introduced via the primer sequences. For L4 complementation the KpnI (FSB184-L4; forward primer) and HindIII (FSB185-L4; reverse primer) restriction sites were introduced via the primer sequences. A BamHI–EcoRV fragment was cloned into pSET152 to obtain pCOM-1213-L2. A KpnI–HindIII fragment was cloned into pMS82 to obtain pCOM-1213-L4. For construction of the control vectors for L2 and L4 complementation analyses the inserts (including the SCO1212 ORF and its promoter region, but not the SCO1213 ORF) were amplified by PCR as follows. Primers FSB184-L2 (forward; BamHI) and FSB200 (reverse; EcoRV) were used for amplifying the control insert for L2. After BamHI/EcoRV digestion the DNA fragment was introduced in pSET152 giving pSET152-SCO1212. Primers FSB184-L4 (forward; KpnI) and FSB201 (reverse; HindIII) were used for amplifying the control insert for L4. After KpnI/ HindIII digestion the DNA fragment was introduced in pMS82 giving pMS82-SCO1212.

**Table 3:** Strains and plasmids used in this work

Bacterial strains	Description	Reference
*E. coli* DH5α	*E. coli* standard strain; F′Ф80 LacZ ∆M15	[29]
*E. coli* ET12567 (pUZ8002)	*E. coli* conjugative strain; *dam, dcm, hsdS, cat, tet*	[30]
*S. coelicolor* M145	*S. coelicolor* parental strain; SCP1^−^ SCP2^−^	[22]
*S. coelicolor* W1	*S. coelicolor* M145 (pVJ-*neo*); Hyg^R^	This work
*S. coelicolor* W2	*S. coelicolor* M145 (pHJH4); Apra^R^	This work
*S. coelicolor* RL-neo	*S. coelicolor* M145 (pRL-*neo*); Hyg^R^	This work
*S. coelicolor* H1-H12	*S. coelicolor* M145 (pVJ-*neo*) suppressor strains; Hyg^R^	This work
*S. coelicolor* L1	*S. coelicolor* M145 (pVJ-*neo*) suppressor strain; Hyg^R^	This work
*S. coelicolor* L2	*S. coelicolor* M145 (pVJ-*neo*) suppressor strain; Hyg^R^	This work
*S. coelicolor* L4	*S. coelicolor* M145 (pHJH4) suppressor strain; Apra^R^	This work
*S. coelicolor* L5	*S. coelicolor* M145 (pHJH4) suppressor strain; Apra^R^	This work
*S. coelicolor* L5-S	*S. coelicolor* L5 (pVJ); Apra^R^, Hyg^R^	This work
*S. coelicolor* L5-S*	*S. coelicolor* L5 (pVJc); Apra^R^, Hyg^R^	This work
*S. coelicolor* WT-S*	*S. coelicolor* M145 (pVJc-*neo*); Hyg^R^	This work
*S. coelicolor* L2–1213	*S. coelicolor* L2 (pCOM-1213-L2); Apra^R^, Hyg^R^	This work
*S. coelicolor* L4–1213	*S. coelicolor* L4 (pCOM-1213-L4); Apra^R^, Hyg^R^	This work
*S. coelicolor* L2-Con	*S. coelicolor* L2 (pSET152-SCO1212); Apra^R^, Hyg^R^	This work
*S. coelicolor* L4-Con	*S. coelicolor* L4 (pMS82-SCO1212); Apra^R^, Hyg^R^	This work
*S. coelicolor* M145-pVJ-1212	*S. coelicolor* M145 (pVJ-1212); Hyg^R^	This work
*S. coelicolor* M145-pVJ-1213	*S. coelicolor* M145 (pVJ-1213); Hyg^R^	This work
*S. coelicolor* M145-pVJc-1212	*S. coelicolor* M145 (pVJc-1212); Hyg^R^	This work
*S. coelicolor* M145-pVJc-1213	*S. coelicolor* M145 (pVJc-1213); Hyg^R^	This work
Plasmids	Description	Reference
pMS82	Integrative vector; phiBT1; Hyg^R^	[31]
pSET152	Integrative vector; phiC31; Apra^R^	[32]
pHJH4	Integrative vector containing *vanJp-neo*; phiC31; Apra^R^	[13]
pVJ	*vanRS*-*vanJ*_prom_ cloned in pMS82; Hyg^R^	This work
pVJ-*neo*	Promoterless *neo* cloned in pVJ; Hyg^R^	This work
pVJc	L5 *vanRS*-*vanJ*_prom_ cloned in pMS82; Hyg^R^	This work
pVJc-*neo*	Promoterless *neo* cloned in pVJc; Hyg^R^	This work
pRL-*neo*	*rpsL*_prom_ cloned in pVJ-*neo*; Hyg^R^	This work
pCOM-1213-L2	SCO1212–13 cloned in pSET152; Apra^R^	This work
pCOM-1213-L4	SCO1212–13 cloned in pMS82; Hyg^R^	This work
pSET152-SCO1212	SCO1212 cloned in pSET152; Apra^R^	This work
pMS82-SCO1212	SCO1212 cloned in pMS82; Hyg^R^	This work
pVJ-1212	Promoterless SCO1212 cloned in pVJ; Hyg^R^	This work
pVJ-1213	Promoterless SCO1213 cloned in pVJ; Hyg^R^	This work
pVJc-1212	Promoterless SCO1212 cloned in pVJc; Hyg^R^	This work
pVJc-1213	Promoterless SCO1213 cloned in pVJc; Hyg^R^	This work

BD Tryptic Soy Broth plus 1.5% agar (TSA) and Difco Nutrient Agar (DifcoNA) were used as standard solid media for growth of the *Streptomyces* strains. BD Tryptic Soy Broth is a general purpose medium that contains (per Liter): 17 g Bacto™ Tryptone (Pancreatic Digest of Casein), 3 g Bacto Soytone (Peptic Digest of Soybean Meal), 2.5 g Glucose, 5 g Sodium Chloride and 2.5 g K_2_HPO_4_ (pH 7.3). Difco Nutrient Agar is a moderately rich, general purpose, solid medium that contains (per Liter): 3 g Beef extract, 5 g Peptone and 1.5% Agar (pH 6.8).

### Vancomycin and teicoplanin susceptibility tests

The MIC (minimum inhibitory concentration) represents the value at which all the isolates in a test population are inhibited. The MIC_50_ represents the value at which ≥50% of the isolates in a test population are inhibited. These values were determined by spreading a confluent lawn of spores onto the medium containing a range of the antibiotic. The number of growing colonies was evaluated after 4 days of incubation at 30 °C. For the rest of the analyses the different antibiotics and compounds were added to the medium before the spore inoculation.

### Growth determination in 96-well plates

*S. coelicolor* W1 cultures were performed in TSA using polystyrene sterile 96-well plates. To each well (containing 100 μl of medium) was added 5 μl of a stock dilution containing 10^6^ spores. Plates were then incubated at 30 °C during 52 h. Growth was determined by optical density (420 nm) using a BMG Fluostar Optima fluorometer.

### Illumina analysis

Genomic DNA was extracted from *S. coelicolor* cells using the “salting-out” method described in Kieser et al. [22]. The extracted DNA was further purified using the DNeasy Mini Spin Columns of the Qiagen Blood & Tissue Kit. The quality of DNA and ratio of absorbance (A260/A280; A260/A230) was checked using a NanoDrop™ spectrophotometer.

A Qubit™ fluorometer was used in association with both the Qubit™ dsDNA Broad Range and High Sensitivity kits to dilute the purified sample DNA in pure water to 0.2 ng per μl. An Illumina® Nextera -XT kit library prep kit was then used to prepare the DNA from each *Streptomyces* strain for sequencing. 1 ng of DNA (in 5 μl) from each strain was used as starting material for this process. Tagmentation of the DNA within the sample was carried out to fragment the DNA and add Illumina® adapters. This was followed by a PCR step, to amplify fragments and add the indexes for sample multiplexing, and a bead-based clean up step. The Agilent® BioAnalyzer was used with the High Sensitivity DNA kit to check the quality and fragment lengths of the DNA during processing. Most of the strains were sequenced twice using different Indexes to provide adequate coverage across each genome. Bead-based normalization was carried out and then samples were pooled and denatured before loading onto the Illumina® MiSeq V3 cartridge ready for sequencing. 2 × 300 paired end sequencing was carried out using the 600 cycle V3 kit.

### Data analysis

The Illumina Miseq raw sequence paired-end fastq files were assessed for sequence quality using the FastQC (https://www.bioinformatics.babraham.ac.uk/projects/fastqc/) program. FastQC provides a simple way to perform quality control checks on raw sequence data, coming from high throughput sequencing pipelines to assess quality phred scores of sequencing data. The raw fastq files were trimmed to remove base pairs from the fastq reads that were deemed low sequence quality based on phred scores; with a phred score cut off threshold of 20 from the 3′ end of fastq reads, using the program Trimomatic [23]. The trimmed paired end fastq reads from each of the strains sequenced were mapped against the reference genome strain *S. coelicolor* M145 using the aligner bowtie 2 [24]. The sam files were converted to a binary bam file format using the program SAMtools [25]. To detect SNP (Single Nucleotide Polymorphisms) and Indels (Insertions Deletions) the program VarScan [26] was used. VarScan uses a robust heuristical and statistical approach to call variants that meet desired thresholds for read depth, base quality and statistical significance. A reference based comparison method was employed using VarScan and the aligned bam files generated from SAMtools. The reference strain *S. coelicolor* M145 was used to detect SNPs/Indels with the VarScan program. To visualize each strain and to look at both the mapped reads from the bam files and SNP and Indels from the sequenced strains, IGV: Integrative Genomics Viewer [27] was used. The vcf files, which are generated using VarScan, allowed visualizing both SNPs and Indels using IGV genome viewer. In-house analysis methods were developed to compare SNPs and Indels between strains.

### Sanger sequencing

For Sanger sequencing fragments up to ~ 3 kbp were amplified by PCR using as template total DNA from the different strains. Then, primers were designed to hybridize within the respective amplification products in order to cover the sequencing of the entire product. To distinguish between the sequences of the chromosomal *vanSR* genes and those contained in the pVJ-*neo* vector two different amplification products were performed as follows: a) primers FSB135-FSB136 (hybridizing in the *S. coelicolor* chromosome) were used to amplified a 2177 bp fragment encompassing just the chromosomal *vanSR* genes; b) primers FSB90-FSB101 (hybridizing in the pMS82 vector) were used to amplified a 3055 bp fragment encompassing the *vanSR* genes and the *vanJ* promoter of pVJ-*neo* vector. Sequencing data was analysed using the ContigExpress program launched from Vector NTI Advance 10 (Invitrogen).

### Time-lapse microscopy

The spores were loaded into B04A microfluidic plates (ONIX, CellASIC) at 4 psi for 30 s, washed at 3 psi for 30 s and grown at constant media flow rate of 2 psi. In all cases TSB (Tryptic Soy Broth) was used as liquid medium. The images were acquired using a Nikon Ti microscope equipped with a Nikon Plan Apo × 100/1.4 oil objective and FRAP-AI v. 7.7.5.0 software (MAG Biosystems, Molecular Devices). Images were acquired every 10 mins during 20 h and the movies were processed using ImageJ (NIH) software. In all cases the videos show bright field channel images displayed at 4 frames per second.

## Additional files

Additional file 1: **Figure S1.** SNP of *vanS* in L5 identified by both Illumina and Sanger sequencing. **Figure S2.** SNP of the *vanS* chromosomal gene in L1 identified by Sanger sequencing. **Figure S3.** SNP of SCO4474 in H5 identified by both Illumina and Sanger sequencing. **Figure S4.** Insertion in SCO3167 of H11 identified by both Illumina and Sanger sequencing. **Figure S5.** SNP of SCO1213 in L2 identified by both Illumina and Sanger sequencing. **Figure S6.** SNP of SCO1213 in L4 identified by both Illumina and Sanger sequencing. **Figure S7.** Sanger sequencing control reactions for the SCO1213 mutations in W1 and W2. **Figure S8.** Sequences of wild type and mutated VanS proteins and alignment of the two sequences. **Figure S9.** Sequences of wild type and mutated SCO1213 proteins and alignment of the two sequences. The image at the bottom shows the localization of the mutation of L2 in SCO1213 and the catalytic triad of the protein according to the CDD: NCBI’s conserved domain database [28]. **Figure S10.** PSIPRED prediction of SCO1213 protein products in WT and L2 strains (http://bioinf.cs.ucl.ac.uk/psipred/). **Figure S11.** STRING analysis of the SCO1212-SCO1213 gene cooccurrence (https://string-db.org/). The figure shows genes families whose occurrence patterns across genomes show similarities. (PDF 1040 kb)

Additional file 2: **Table S1.** Analysis of the growth of L2 and L4 complemented strains grown in DifcoNA (with or without 1% K_2_HPO_4_ addition) and different concentrations of lysozyme, vancomycin and teicoplanin. The concentration of the compounds is shown as μg per mL. (DOCX 16 kb)

Additional file 3: **Table S2.** Analysis of the effect of coupling SCO1212 and SCO1213 to pVJ and pVJc on the growth of the next strains: SC100 (*S. coelicolor* M145-pVJ-1212; isolate 1), SC101 (*S. coelicolor* M145-pVJ-1212; isolate 2), SC102 (*S. coelicolor* M145-pVJc-1212; isolate 1), SC103 (*S. coelicolor* M145-pVJc-1212; isolate 2), SC104 (*S. coelicolor* M145-pVJ-1213; isolate 1), SC105 (*S. coelicolor* M145-pVJ-1213; isolate 2), SC106 (*S. coelicolor* M145-pVJc-1213; isolate 1), SC107 (*S. coelicolor* M145-pVJc-1213; isolate 2) in DifcoNA (with or without 1% K_2_HPO_4_ addition) and different concentrations of lysozyme, vancomycin and teicoplanin. The concentration of the compounds is shown as μg per mL. (DOCX 15 kb)

Additional file 4: **Video S1.** Spores of W1 grown in the CellASIC ONIX microfluidic chamber in the presence of TSB medium. The images were acquired every 10 mins during 20 h. The video shows bright field images displayed at 4 frames per second. (AVI 16079 kb)

Additional file 5: **Video S2.** Spores of W2 grown in the CellASIC ONIX microfluidic chamber in the presence of TSB medium. The images were acquired every 10 mins during 20 h. The video shows bright field images displayed at 4 frames per second. (AVI 18898 kb)

Additional file 6: **Video S3.** Spores of L1 grown in the CellASIC ONIX microfluidic chamber in the presence of TSB medium. The images were acquired every 10 mins during 20 h. The video shows bright field images displayed at 4 frames per second. (AVI 15541 kb)

Additional file 7: **Video S4.** Spores of L2 grown in the CellASIC ONIX microfluidic chamber in the presence of TSB medium. The images were acquired every 10 mins during 20 h. The video shows bright field images displayed at 4 frames per second. (AVI 28754 kb)

Additional file 8: **Video S5.** Spores of L4 grown in the CellASIC ONIX microfluidic chamber in the presence of TSB medium. The images were acquired every 10 mins during 20 h. The video shows bright field images displayed at 4 frames per second. (AVI 29210 kb)

Additional file 9: **Video S6.** Spores of L5 grown in the CellASIC ONIX microfluidic chamber in the presence of TSB medium. The images were acquired every 10 mins during 20 h. The video shows bright field images displayed at 4 frames per second. (AVI 14438 kb)

Additional file 10: **Table S3.** Primers used in this work. (DOCX 16 kb)

## Abbreviations

AMR: Antimicrobial resistance
D-Ala: D-Alanine
DifcoNA: Difco Nutrient Agar
D-Lac: D-Lactate
MIC: Minimum inhibitory concentration
MRSA: Methicillin-resistant *Staphylococcus aureus*
Pi: Inorganic phosphate
SNP: Single nucleotide polymorphism
VR: Vancomycin resistance

## References

[1] Chang VS, Dhaliwal DK, Raju L, Kowalski RP (2015). Antibiotic resistance in the treatment of *Staphylococcus aureus* keratitis: a 20-year review. Cornea.

[2] Cattoir V, Leclercq R (2013). Twenty-five years of shared life with vancomycin-resistant enterococci: is it time to divorce?. J Antimicrob Chemother.

[3] Rossolini GM, Mantengoli E, Montagnani F, Pollini S (2010). Epidemiology and clinical relevance of microbial resistance determinants versus anti-gram-positive agents. Curr Op Microbiol.

[4] Leclercq R, Derlot E, Duval J, Courvalin P (1988). Plasmid-mediated resistance to vancomycin and teicoplanin in *Enterococcus faecium*. N Engl J Med.

[5] Hong HJ, Hutchings MI, Neu JM, Wright GD, Paget MS, Buttner MJ (2004). Characterization of an inducible vancomycin resistance system in *Streptomyces coelicolor* reveals a novel gene (*vanK*) required for drug resistance. Mol Microbiol.

[6] D'Costa VM, King CE, Kalan L, Morar M, Sung WW, Schwarz C, Froese D, Zazula G, Calmels F, Debruyne R (2011). Antibiotic resistance is ancient. Nature.

[7] Hopwood DA (2007). *Streptomyces* in nature and medicine.

[8] Reynolds PE, Courvalin P (2005). Vancomycin resistance in enterococci due to synthesis of precursors terminating in D-Alanyl-D-serine. Antimicrob Agents Chemother.

[9] Hong H-J, Hutchings MI, Hill LM, Buttner MJ (2005). The role of the novel fem protein VanK in vancomycin resistance in *Streptomyces coelicolor*. J Biol Chem.

[10] Novotna G, Hill C, Vincent K, Liu C, Hong HJ (2012). A novel membrane protein, VanJ, conferring resistance to teicoplanin. Antimicrob Agents Chemother.

[11] Santos-Beneit F, Ordóñez-Robles M, Martín JF (2017). Glycopeptide resistance: links with inorganic phosphate metabolism and cell envelope stress. Biochem Pharmacol.

[12] Santos-Beneit F, Martín JF (2013). Vancomycin resistance in *Streptomyces coelicolor* is phosphate-dependent but is not mediated by the PhoP regulator. J Global Antimicrob Resist.

[13] Kwun MJ, Novotna G, Hesketh AR, Hill L, Hong HJ (2013). In vivo studies suggest that induction of VanS-dependent vancomycin resistance requires binding of the drug to D-ala-D-ala termini in the peptidoglycan cell-wall. Antimicrob Agents Chemother.

[14] Figueiredo TA, Sobral RG, Ludovice AM, Almeida JM, Bui NK, Vollmer W, de Lencastre H, Tomasz A (2012). Identification of genetic determinants and enzymes involved with the amidation of glutamic acid residues in the peptidoglycan of *Staphylococcus aureus*. PLoS Pathog.

[15] Münch D, Roemer T, Lee SH, Engeser M, Sahl HG, Schneider T (2012). Identification and in vitro analysis of the GatD/MurT enzyme-complex catalyzing lipid II amidation in *Staphylococcus aureus*. PLoS Pathog.

[16] Bryson K, McGuffin LJ, Marsden RL, Ward JJ, Sodhi JS, Jones DT (2005). Protein structure prediction servers at University College London. Nucleic Acids Res.

[17] Santos-Beneit F, Fernández-Martínez LT, Rodríguez-García A, Martín-Martín S, Ordóñez-Robles M, Yagüe P, Manteca A, Martín JF (2014). Transcriptional response to vancomycin in a highly vancomycin-resistant *Streptomyces coelicolor* mutant. Future Microbiol.

[18] Hutchings MI, Hong HJ, Buttner MJ (2006). The vancomycin resistance VanRS two-component signal transduction system of *Streptomyces coelicolor*. Mol Microbiol.

[19] Bui NK, Eberhardt A, Vollmer D, Kern T, Bougault C, Tomasz A, Simorre JP, Vollmer W (2012). Isolation and analysis of cell wall components from *Streptococcus pneumoniae*. Anal Biochem.

[20] Leprohon P, Gingras H, Ouennane S, Moineau S, Ouellette M (2015). A genomic approach to understand interactions between Streptococcus pneumoniae and its bacteriophages. BMC Genomics.

[21] Münch D, Engels I, Müller A, Reder-Christ K, Falkenstein-Paul H, Bierbaum G, Grein F, Bendas G, Sahl HG, Schneider T (2015). Structural variations of the cell wall precursor lipid II and their influence on binding and activity of the lipoglycopeptide antibiotic oritavancin. Antimicrob Agents Chemother.

[22] Kieser T, Bibb MJ, Buttner MJ, Chater KF, Hopwood DA. A Practical Guide to *Streptomyces* Genetics. Norwich: John Innes Foundation; 2000.

[23] Bolger AM, Lohse M, Usadel B (2014). Trimmomatic: a flexible trimmer for Illumina sequence data. Bioinformatics.

[24] Langmead B, Salzberg S (2012). Fast gapped-read alignment with bowtie 2. Nat Methods.

[25] Li H, Handsaker B, Wysoker A, Fennell T, Ruan J, Homer N, Marth G, Abecasis G, Durbin R, 1000 Genome Project Data Processing Subgroup (2009). The sequence alignment/map format and SAMtools. Bioinformatics.

[26] Koboldt DC, Chen K, Wylie T, Larson DE, McLellan MD, Mardis ER, Weinstock GM, Wilson RK, Ding L (2009). VarScan: variant detection in massively parallel sequencing of individual and pooled samples. Bioinformatics.

[27] Robinson JT, Thorvaldsdóttir H, Winckler W, Guttman M, Lander ES, Getz G, Mesirov JP (2011). Integrative genomics viewer. Nat Biotechnol.

[28] Marchler-Bauer A, Derbyshire MK, Gonzales NR, Lu S, Chitsaz F, Geer LY, Geer RC, He J, Gwadz M, Hurwitz DI (2015). CDD: NCBI's conserved domain database. Nucleic Acids Res.

[29] Hanahan D (1983). Studies on transformation of *Escherichia coli* with plasmids. J Mol Biol.

[30] MacNeil DJ, Occi JL, Gewain KM, MacNeil T, Gibbons PH, Ruby CL, Danis SJ (1992). Complex organization of the *Streptomyces avermitilis* genes encoding the avermectin polyketide synthase. Gene.

[31] Gregory MA, Till R, Smith MC (2003). Integration site for *Streptomyces* phage phiBT1 and development of site-specific integrating vectors. J Bacteriol.

[32] Bierman M, Logan R, O'Brien K, Seno ET, Rao RN, Schoner BE (1992). Plasmid cloning vectors for the conjugal transfer of DNA from *Escherichia coli* to *Streptomyces* spp. Gene.

