# Supplementary material for: Genome sequencing analysis of Streptomyces coelicolor mutants that overcome the phosphate-depending vancomycin lethal effect
Source: BMC Genomics. 2018 Jun 14;19:457. doi: 10.1186/s12864-018-4838-z (PMC6001138; doi:10.1186/s12864-018-4838-z)
Supplement: Supplementary file 1 — Figure S1. SNP of vanS in L5 identified by both Illumina and Sanger sequencing. Figure S2. SNP of the vanS chromosomal gene in L1 identified by Sanger sequencing. Figure S3. SNP of SCO4474 in H5 identified by both Illumina and Sanger sequencing. Figure S4. Insertion in SCO3167 of H11 identified by both Illumina and Sanger sequencing. Figure S5. SNP of SCO1213 in L2 identified by both Illumina and Sanger sequencing. Figure S6. SNP of SCO1213 in L4 identified by both Illumina and Sanger sequencing. Figure S7. Sanger sequencing control reactions for the SCO1213 mutations in W1 and W2. Figure S8. Sequences of wild type and mutated VanS proteins and alignment of the two sequences. Figure S9. Sequences of wild type and mutated SCO1213 proteins and alignment of the two sequences. The image at the bottom shows the localization of the mutation of L2 in SCO1213 and the catalytic triad of the protein according to the CDD: NCBI’s conserved domain database [28]. Figure S10. PSIPRED prediction of SCO1213 protein products in WT and L2 strains (http://bioinf.cs.ucl.ac.uk/psipred/). Figure S11. STRING analysis of the SCO1212-SCO1213 gene cooccurrence (https://string-db.org/). The figure shows genes families whose occurrence patterns across genomes show similarities. (PDF 1040 kb) [file 12864_2018_4838_MOESM1_ESM.pdf]

<u>SNP of *vanS* in L5 (identified by both Illumina and Sanger sequencing)</u>

Sanger

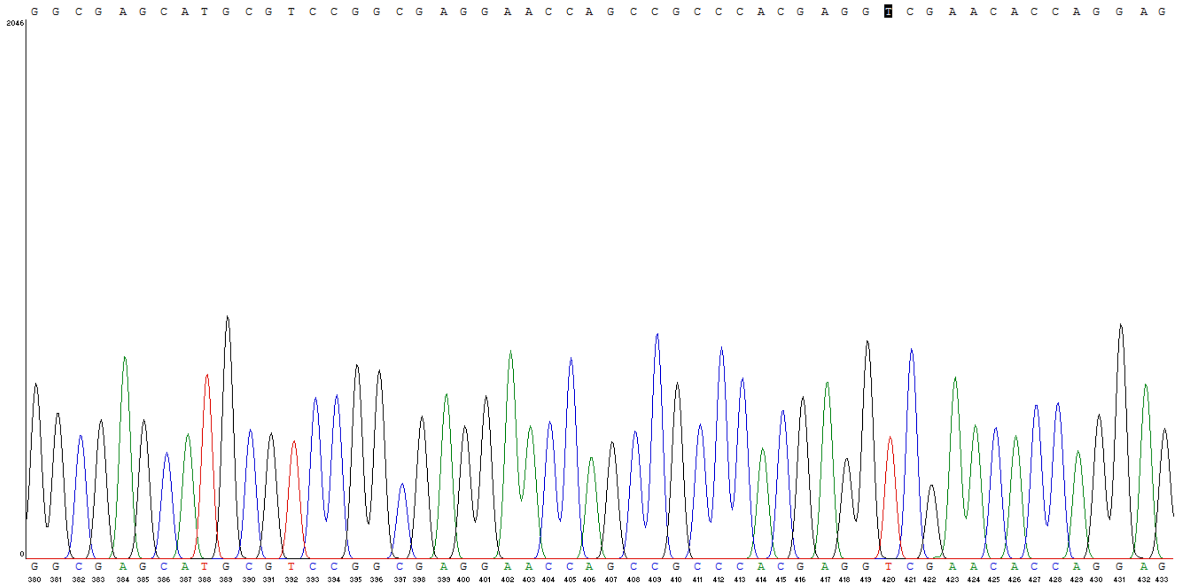

Illumina

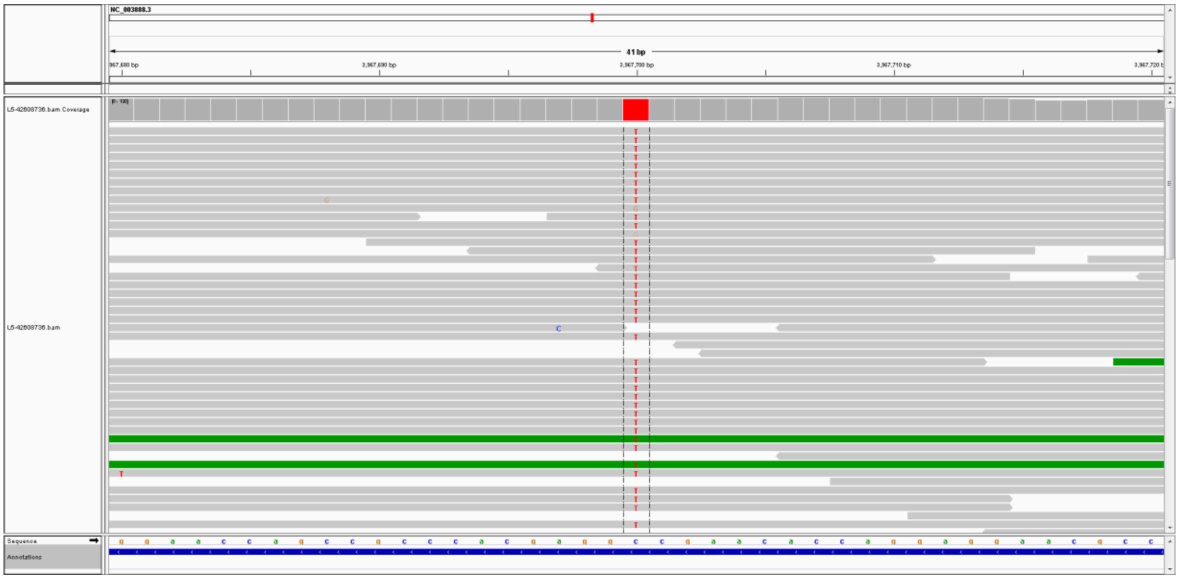

Supplementary Figure S1.

<u>SNP of the *vanS* chromosomal gene in L1 (identified by Sanger sequencing)</u>

Sanger reaction (L1 strain): sequences shown are from 2 different PCR reactions

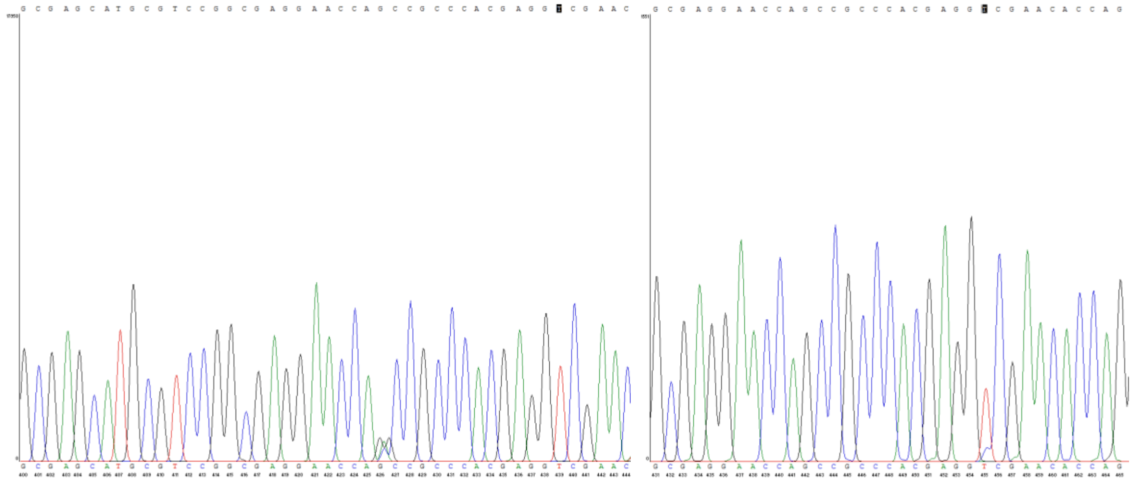

Sanger reaction (W1 strain)

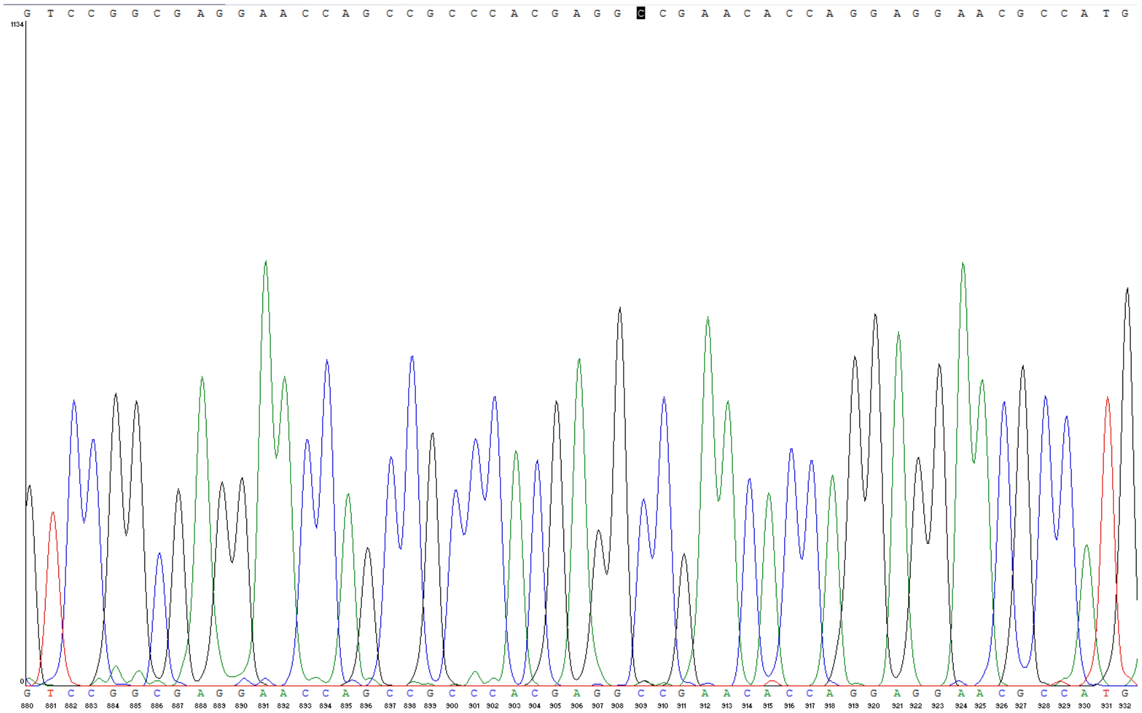

Supplementary Figure S2.

SNP of SCO4474 in H5 (identified by both Illumina and Sanger sequencing)

Sanger

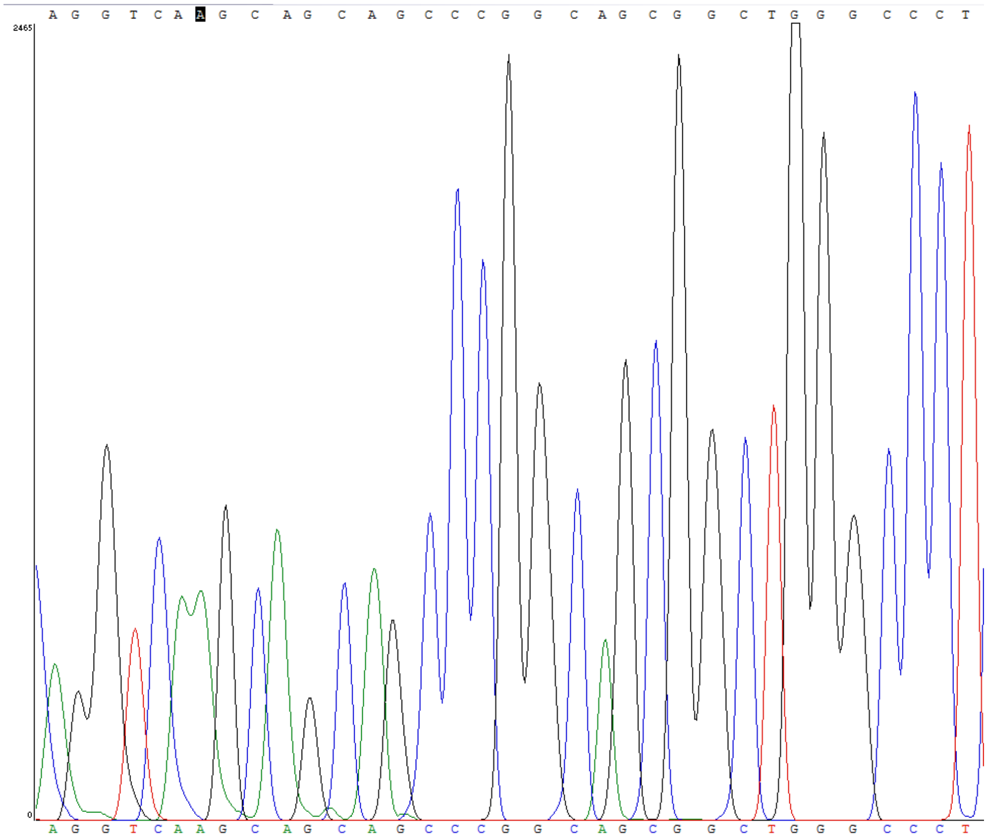

Illumina

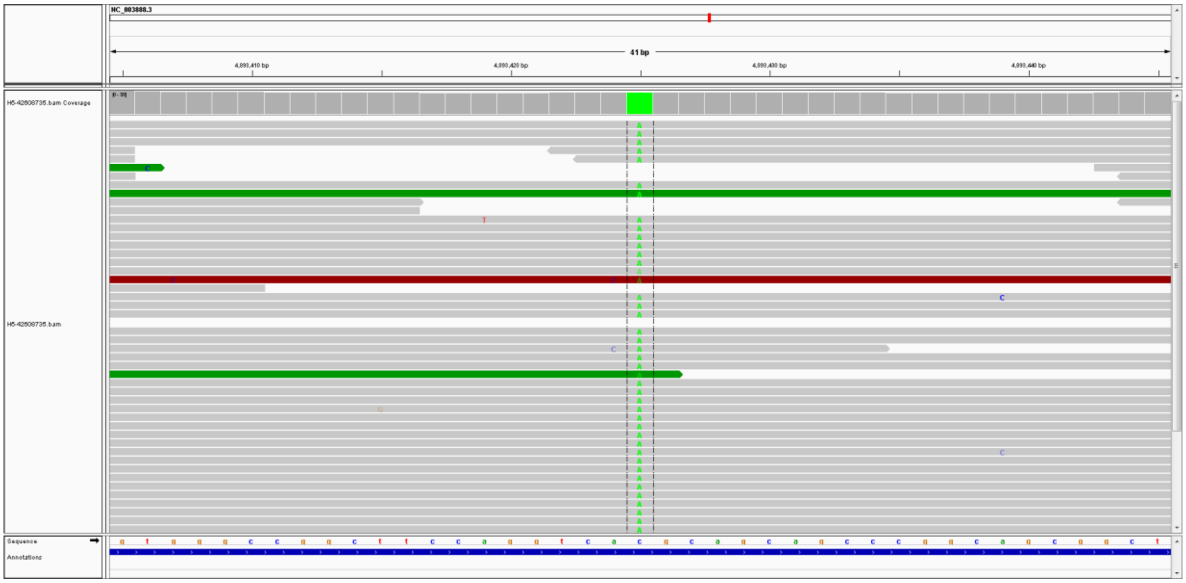

Supplementary Figure S3.

Insertion in SCO3167 of H11 (identified by both Illumina and Sanger sequencing)

Sanger

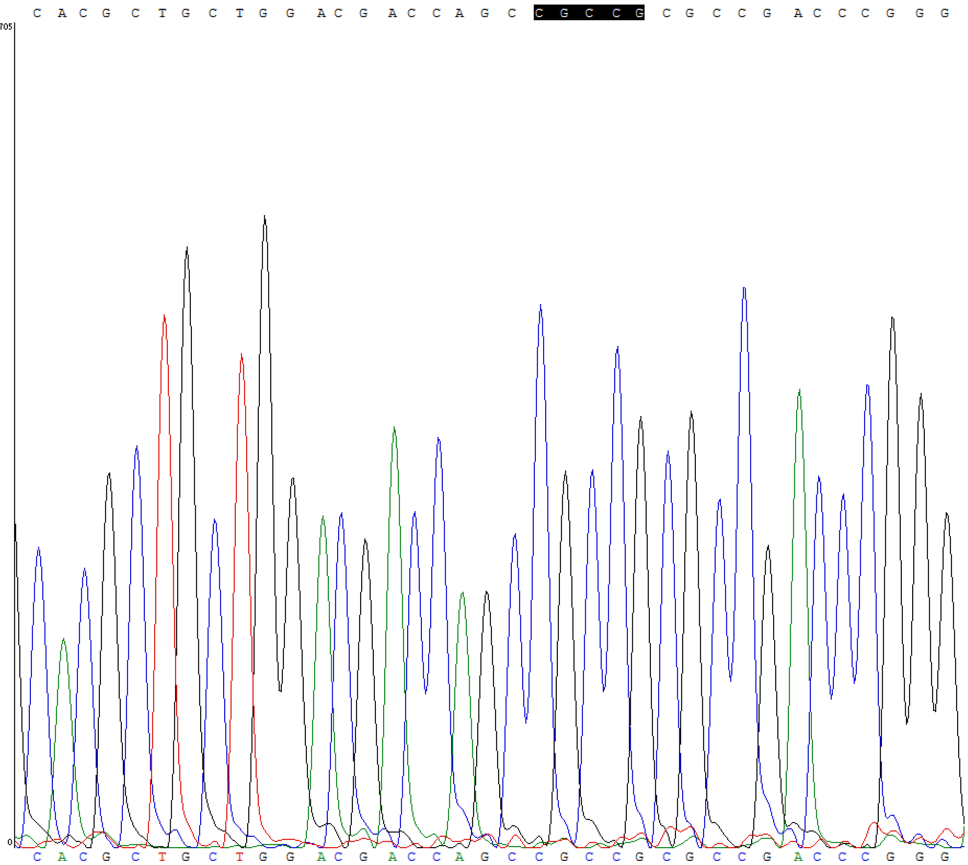

Illumina

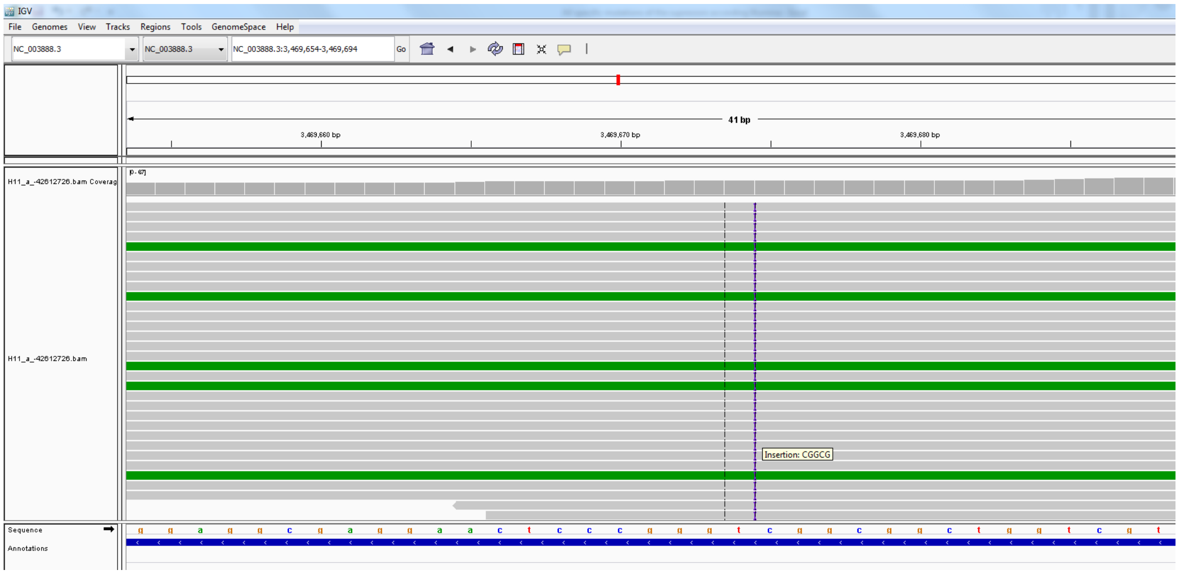

Supplementary Figure S4.

SNP of SCO1213 in L2 (identified by both Illumina and Sanger sequencing)

Sanger

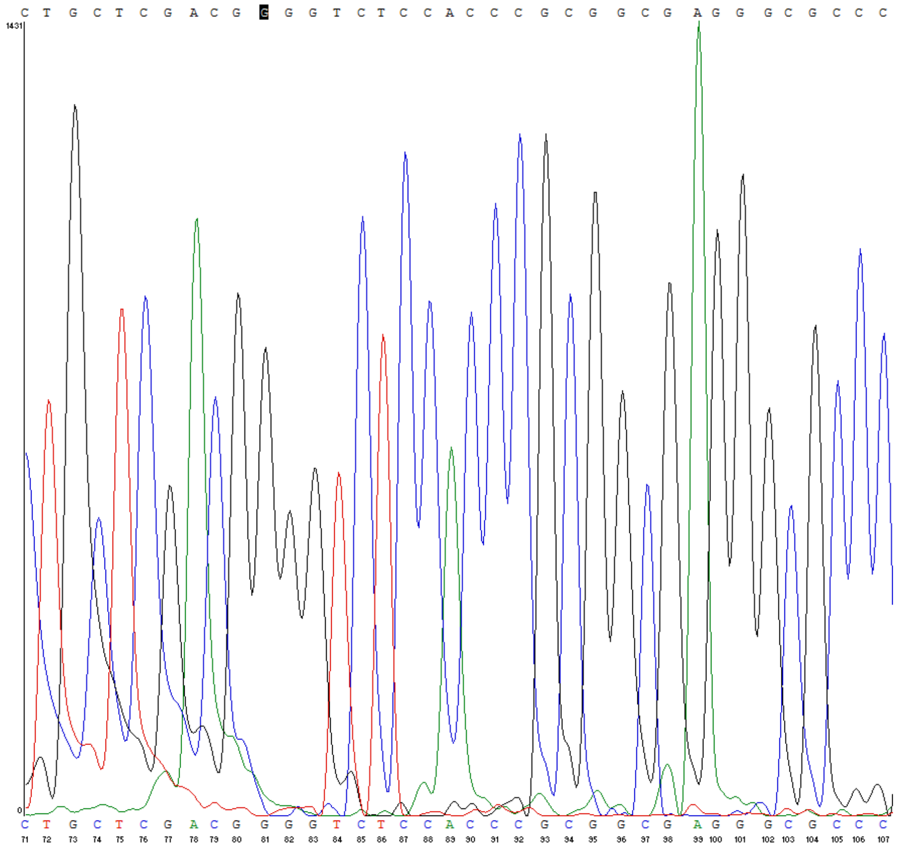

Illumina

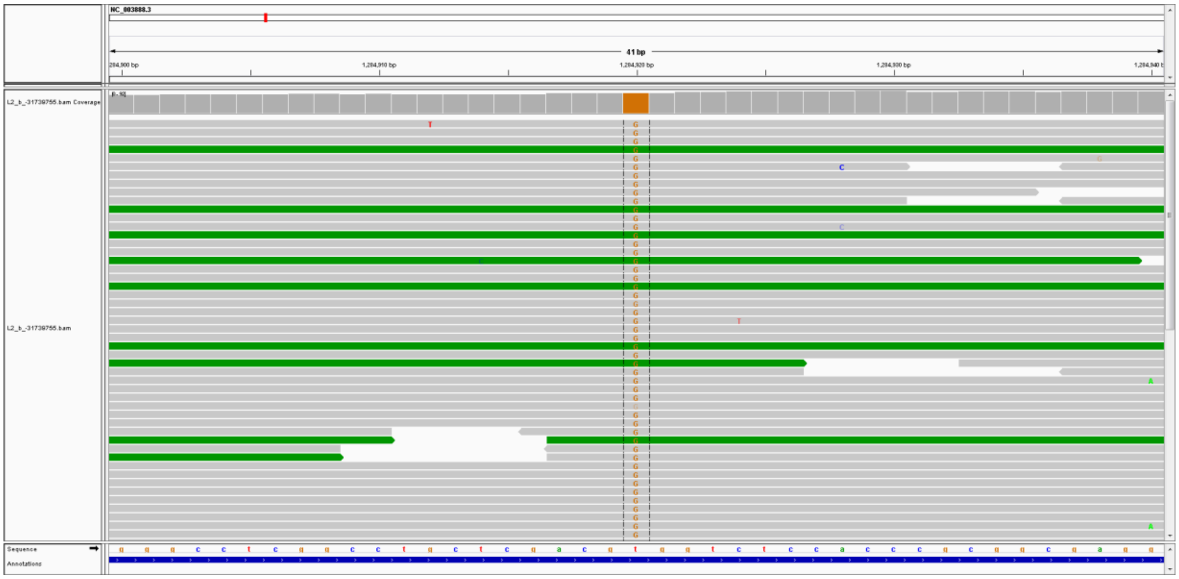

Supplementary Figure S5.

<u>**SNP of SCO1213 in L4 (identified by both Illumina and Sanger sequencing)**</u>

Sanger

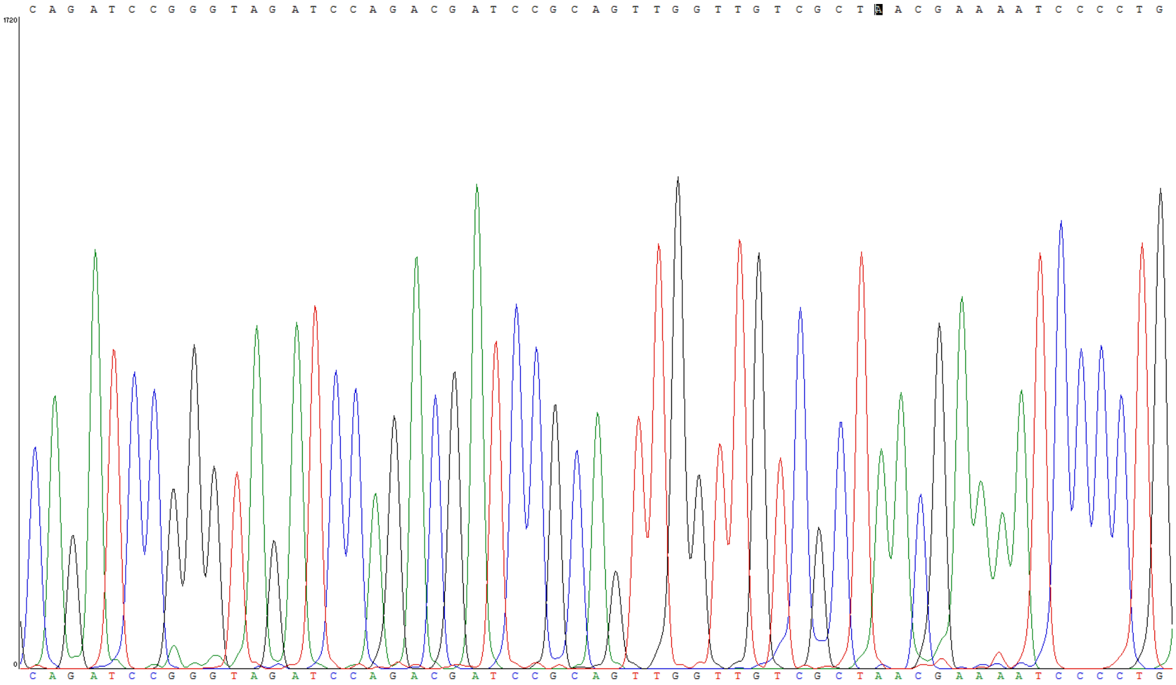

Illumina

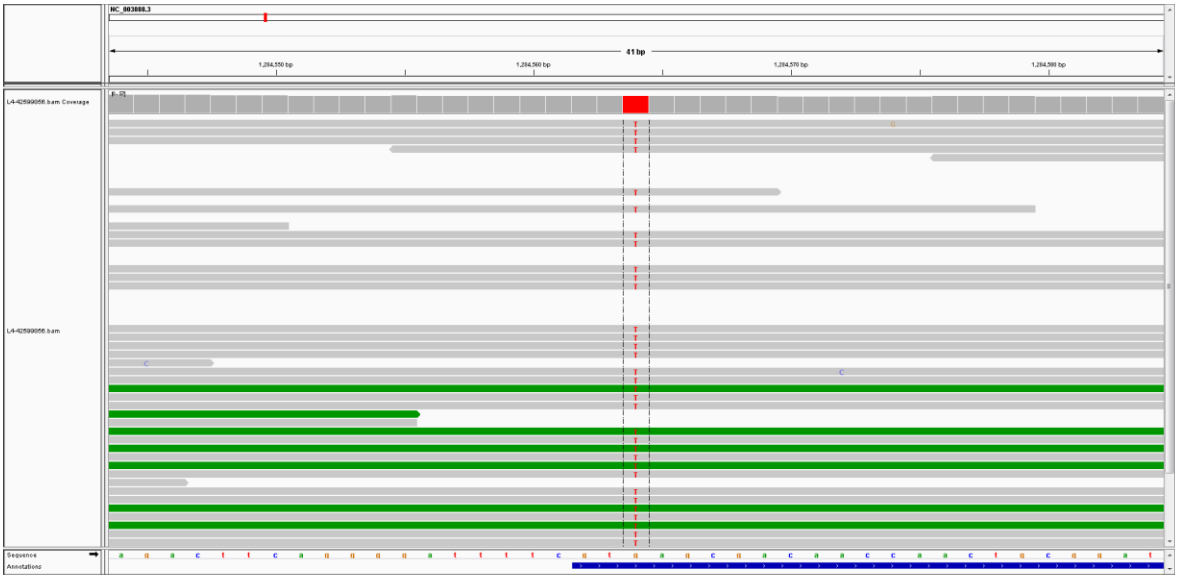

Supplementary Figure S6.

No variant of SCO1213 in W1 (identified by both Illumina and Sanger sequencing)

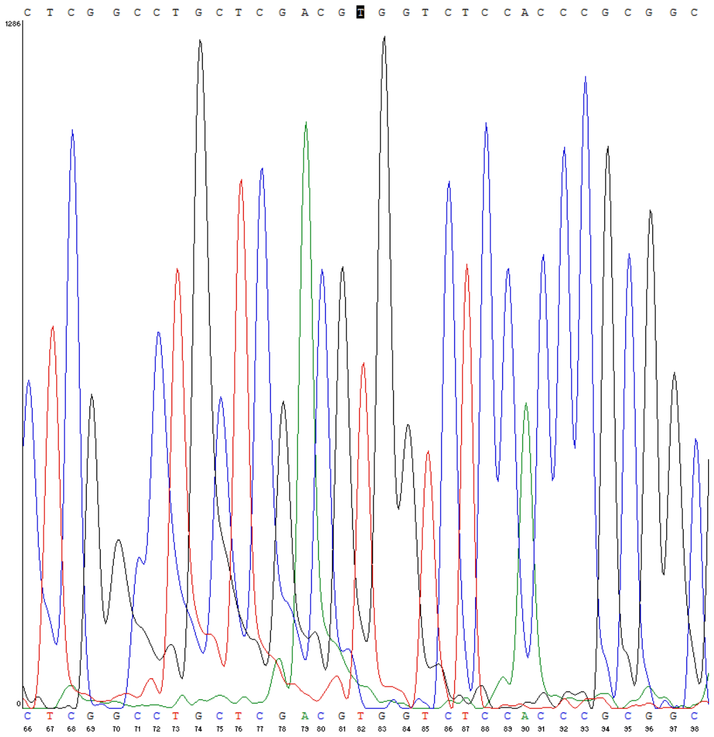

No variant of SCO1213 in W2 (identified by both Illumina and Sanger sequencing)

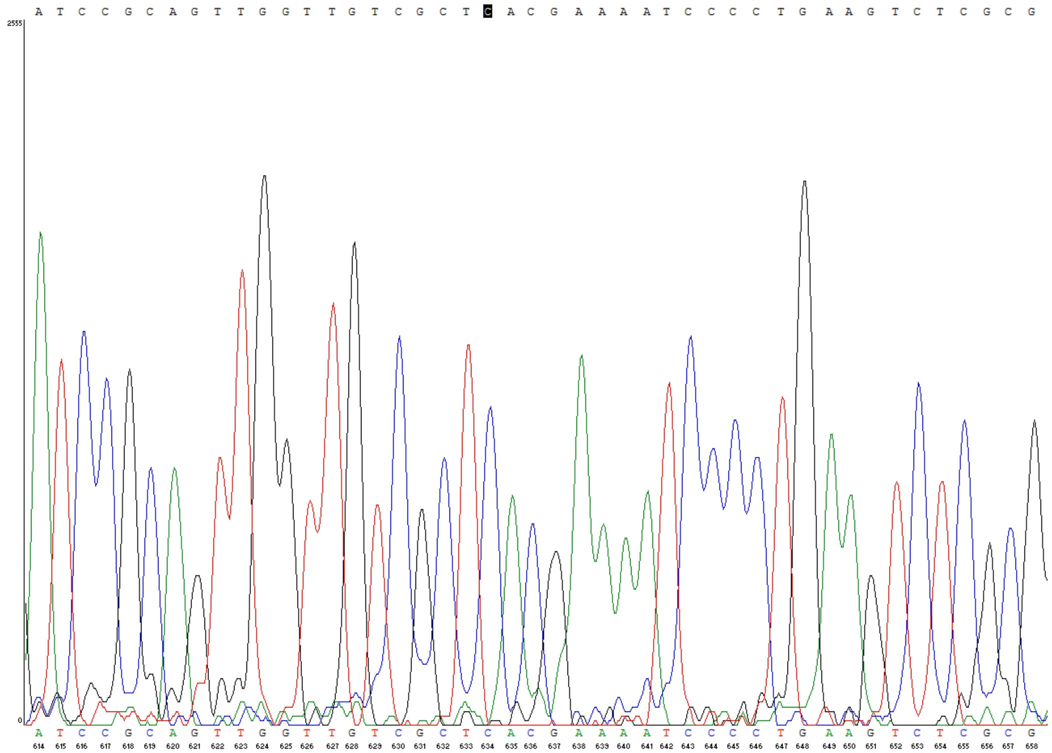

Supplementary Figure S7.

SNP of *vanS* in L1 and L5 (Gly77→Asp77)

WT protein

vdrrpglsvrlkltlsyagfltlagvlllvavgvflldqgwlltnergavratpgtvflrsfaptaawvmafllvf**g**lvggwflagrmlapldrite atrtaatgslshrirlpgrrdeyreladafdemlarleahvaeqrrfaanashelrtplavskaildvartdphqdpgeiidrlhavntraidlteall llsragqrsftreqvdmsllaeeatetllpfaekhgvtletrghvtlalgspalllqlttnlvhnaivhnlpgrgrvwihtaagprttrlvventgdli sphqastltepfqrgterihtdhpgvglglaivntitqahdgtltltprhsgglrvtvelpaaaphtgr*

Mut protein

vdrrpglsvrlkltlsyagfltlagvlllvavgvflldqgwlltnergavratpgtvflrsfaptaawvmafllvf**d**lvggwflagrmlapldrite atrtaatgslshrirlpgrrdeyreladafdemlarleahvaeqrrfaanashelrtplavskaildvartdphqdpgeiidrlhavntraidlteall llsragqrsftreqvdmsllaeeatetllpfaekhgvtletrghvtlalgspalllqlttnlvhnaivhnlpgrgrvwihtaagprttrlvventgdli sphqastltepfqrgterihtdhpgvglglaivntitqahdgtltltprhsgglrvtvelpaaaphtgr*

```
  1 vdrrpglsvrlkltlsyagfltlagvlllvavgvflldqgwlltnergav    50
    ||||||||||||||||||||||||||||||||||||||||||||||||||
  1 vdrrpglsvrlkltlsyagfltlagvlllvavgvflldqgwlltnergav    50

 51 ratpgtvflrsfaptaawvmafllvfglvggwflagrmlapldriteatr   100
    ||||||||||||||||||||||||||.|||||||||||||||||||||||
 51 ratpgtvflrsfaptaawvmafllvfdlvggwflagrmlapldriteatr   100

101 taatgslshrirlpgrrdeyreladafdemlarleahvaeqrrfaanash   150
    ||||||||||||||||||||||||||||||||||||||||||||||||||
101 taatgslshrirlpgrrdeyreladafdemlarleahvaeqrrfaanash   150

151 elrtplavskaildvartdphqdpgeiidrlhavntraidltealllsr   200
    ||||||||||||||||||||||||||||||||||||||||||||||||||
151 elrtplavskaildvartdphqdpgeiidrlhavntraidltealllsr   200

201 agqrsftreqvdmsllaeeatetllpfaekhgvtletrghvtlalgspal   250
    ||||||||||||||||||||||||||||||||||||||||||||||||||
201 agqrsftreqvdmsllaeeatetllpfaekhgvtletrghvtlalgspal   250

251 llqlttnlvhnaivhnlpgrgrvwihtaagprttrlvventgdlisphqa   300
    ||||||||||||||||||||||||||||||||||||||||||||||||||
251 llqlttnlvhnaivhnlpgrgrvwihtaagprttrlvventgdlisphqa   300

301 stltepfqrgterihtdhpgvglglaivntitqahdgtltltprhsgglr   350
    ||||||||||||||||||||||||||||||||||||||||||||||||||
301 stltepfqrgterihtdhpgvglglaivntitqahdgtltltprhsgglr   350

351 vtvelpaaaphtgr*    365
    ||||||||||||||
351 vtvelpaaaphtgr*    365
```

Supplementary Figure S8.

SNP of SCO1213 in L2 (Val120 → Gly120)

WT protein

vsdnqlrivwiypdllstygdqgnalvverrarqrgldvarldvrsdqpiptsgdiylvgggedrpqrlaaerlrrdgglyraven
gaivfsvcagyqilghefindlgqrepglglldvvstrgegarcvgdvlgdidprlglppltgfenhqgvthvgpnarplaqvrfg
ngngtgdgtegayndtvfgtymhgpvlarnpliadlllklaldvnalpptddrwyealrneriaaaqqpa*

Mut protein

vsdnqlrivwiypdllstygdqgnalvverrarqrgldvarldvrsdqpiptsgdiylvgggedrpqrlaaerlrrdgglyraven
gaivfsvcagyqilghefindlgqrepglglldgvstrgegarcvgdvlgdidprlglppltgfenhqgvthvgpnarplaqvrfg
ngngtgdgtegayndtvfgtymhgpvlarnpliadlllklaldvnalpptddrwyealrneriaaaqqpa*

```
  1 vsdnqlrivwiypdllstygdqgnalvverrarqrgldvarldvrsdqpi    50
    ||||||||||||||||||||||||||||||||||||||||||||||||||
  1 vsdnqlrivwiypdllstygdqgnalvverrarqrgldvarldvrsdqpi    50

 51 ptsgdiylvgggedrpqrlaaerlrrdgglyravengaivfsvcagyqil   100
    ||||||||||||||||||||||||||||||||||||||||||||||||||
 51 ptsgdiylvgggedrpqrlaaerlrrdgglyravengaivfsvcagyqil   100

101 ghefindlgqrepglglldvvstrgegarcvgdvlgdidprlglppltgf   150
    |||||||||||||||||||.||||||||||||||||||||||||||||||
101 ghefindlgqrepglglldgvstrgegarcvgdvlgdidprlglppltgf   150

151 enhqgvthvgpnarplaqvrfgngngtgdgtegayndtvfgtymhgpvla   200
    ||||||||||||||||||||||||||||||||||||||||||||||||||
151 enhqgvthvgpnarplaqvrfgngngtgdgtegayndtvfgtymhgpvla   200

201 rnpliadlllklaldvnalpptddrwyealrneriaaaqqpa*    243
    ||||||||||||||||||||||||||||||||||||||||||
201 rnpliadlllklaldvnalpptddrwyealrneriaaaqqpa*    243
```

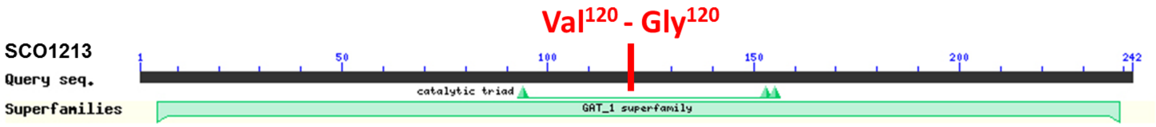

Supplementary Figure S9.

PSIPRED prediction of the mutation of SCO1213 in L2 (Val120 → Gly120)

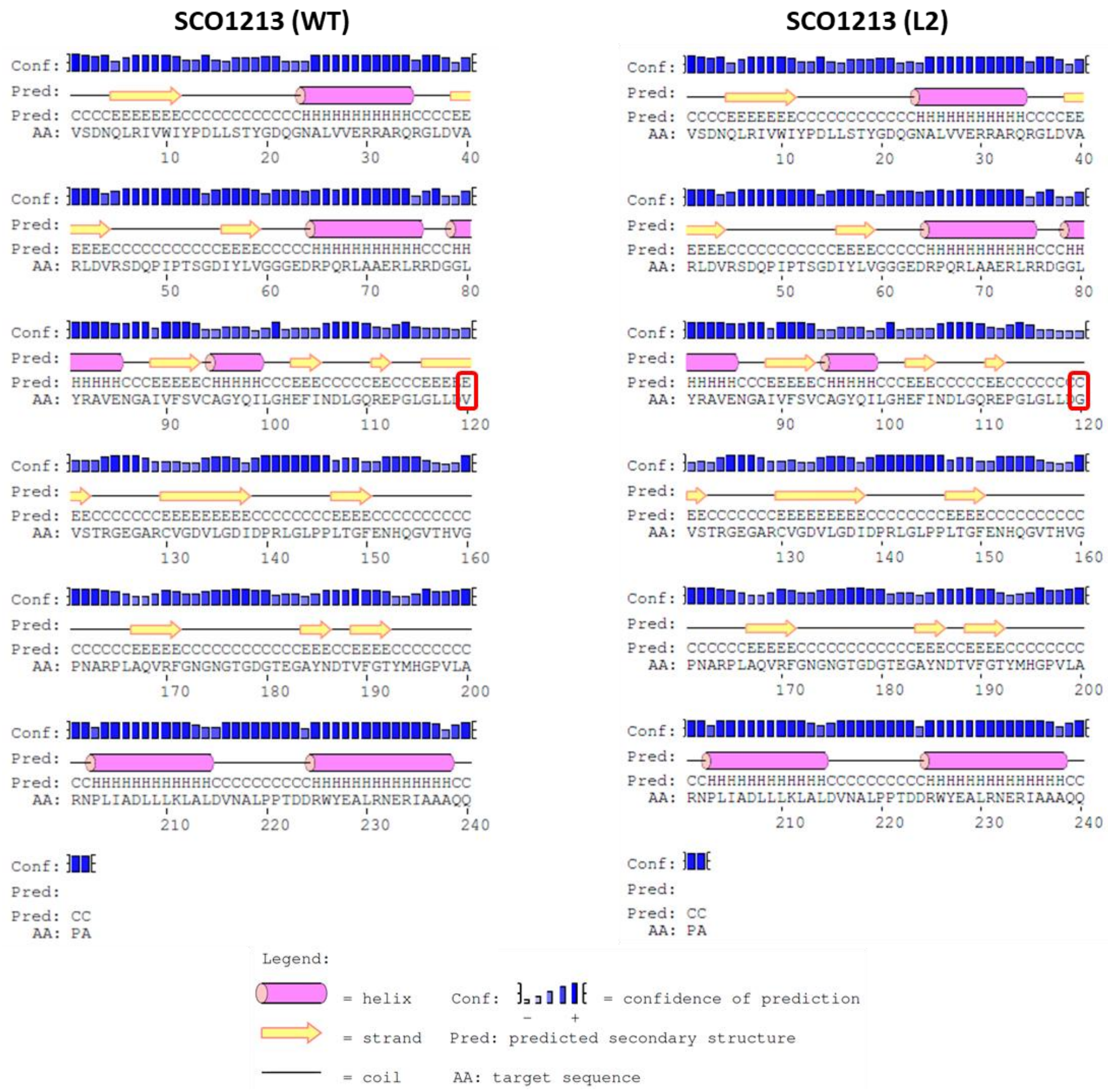

Supplementary Figure S10.

STRING analysis of the SCO1212-SCO1213 gene cooccurrence

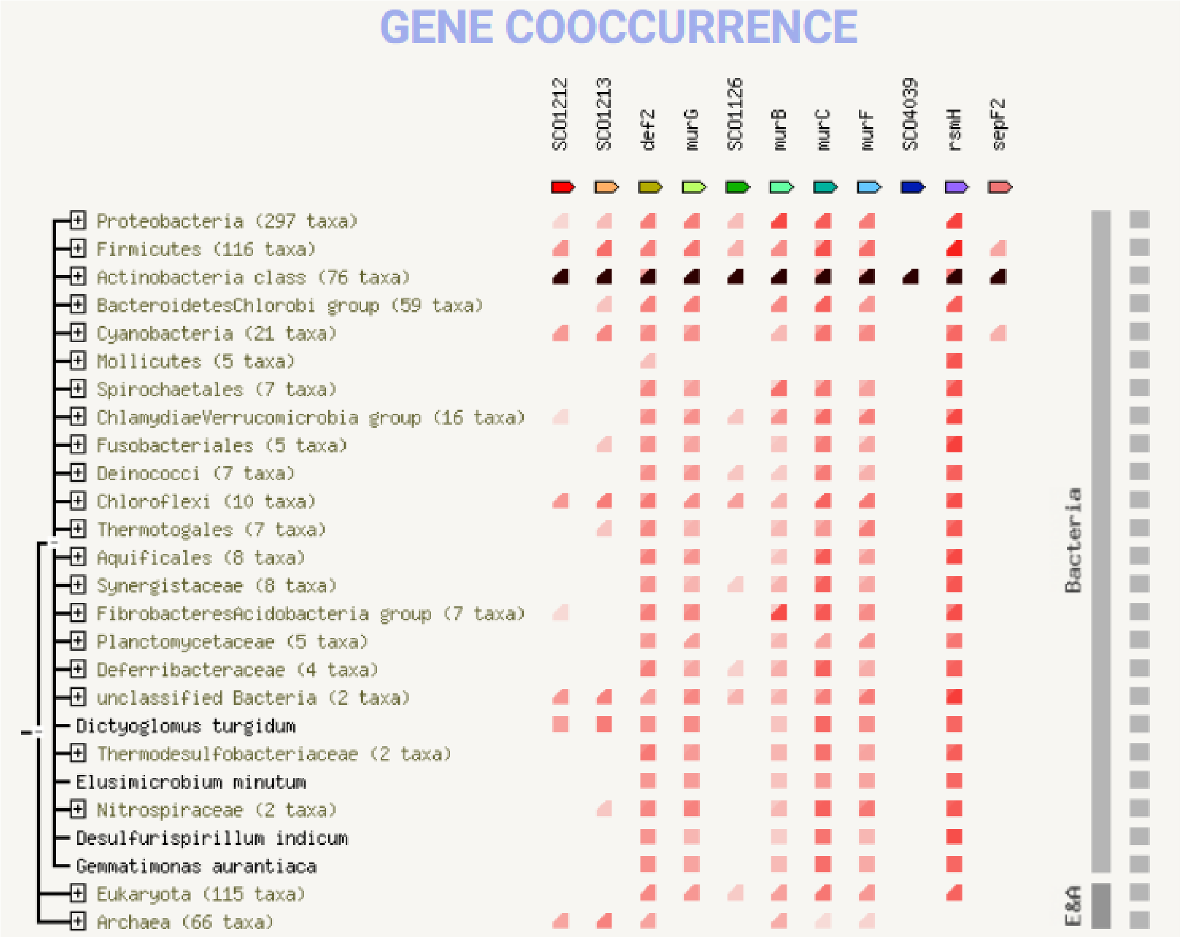

Supplementary Figure S11.
