# Supplementary material for: Genome sequencing analysis of Streptomyces coelicolor mutants that overcome the phosphate-depending vancomycin lethal effect
Source: BMC Genomics. 2018 Jun 14;19:457. doi: 10.1186/s12864-018-4838-z (PMC6001138; doi:10.1186/s12864-018-4838-z)
Supplement: Supplementary file 2 — Table S1. Analysis of the growth of L2 and L4 complemented strains grown in DifcoNA (with or without 1% K2HPO4 addition) and different concentrations of lysozyme, vancomycin and teicoplanin. The concentration of the compounds is shown as μg per mL. (DOCX 16 kb) [file 12864_2018_4838_MOESM2_ESM.docx]

| **DifcoNA** | | |  |  |  |  |  |  |  |  |  |  |  |
| --- | --- | --- | --- | --- | --- | --- | --- | --- | --- | --- | --- | --- | --- |
| **Lys** | **Van** | **Tei** | **W1/W2** | **L2** | **L2-1213_1_** | **L2-1213_2_** | **L2-Con_1_** | **L2-Con_2_** | **L4** | **L4-1213_1_** | **L4-1213_2_** | **L4-Con_1_** | **L4-Con_2_** |
| **-** | **-** | **-** | ++++ | ++++ | ++++ | ++++ | ++++ | ++++ | ++++ | ++++ | ++++ | ++++ | ++++ |
| **-** | **50** | **-** | ++++ | ++++ | ++++ | ++++ | ++++ | ++++ | ++++ | ++++ | ++++ | ++++ | ++++ |
| **-** | **50** | **5** | ++++ | ++++ | ++++ | ++++ | ++++ | ++++ | ++++ | ++++ | ++++ | ++++ | ++++ |
| **10** | **-** | **-** | ++++ | ++++ | ++++ | ++++ | ++++ | ++++ | ++ | ++++ | ++++ | ++ | ++ |
| **100** | **-** | **-** | ++++ | ++ | ++++ | ++++ | ++ | ++ | + | ++++ | ++++ | + | + |
| **500** | **-** | **-** | ++++ | - | ++ | ++ | - | - | - | ++ | ++ | - | - |
| **10** | **50** | **-** | ++++ | + | ++ | ++ | + | + | - | ++++ | ++++ | - | - |
| **100** | **50** | **-** | ++ | - | ++ | ++ | - | - | - | ++ | ++ | - | - |
| **500** | **50** | **-** | ++ | - | ++ | ++ | - | - | - | ++ | ++ | - | - |
| **DifcoNA+1%Pi** | | |  |  |  |  |  |  |  |  |  |  |  |
| **Lys** | **Van** | **Tei** | **W1/W2** | **L2** | **L2-1213_1_** | **L2-1213_2_** | **L2-Con_1_** | **L2-Con_2_** | **L4** | **L4-1213_1_** | **L4-1213_2_** | **L4-Con_1_** | **L4-Con_2_** |
| **-** | **-** | **-** | ++++ | ++++ | ++++ | ++++ | ++++ | ++++ | ++++ | ++++ | ++++ | ++++ | ++++ |
| **-** | **50** | **-** | - | ++++ | + | - | ++ | ++ | ++++ | - | - | ++ | ++ |
| **-** | **50** | **5** | - | ++++ | - | - | ++ | ++ | ++++ | - | - | ++ | ++ |
| **10** | **-** | **-** | ++++ | - | ++++ | ++++ | - | - | - | ++++ | ++++ | - | - |
| **100** | **-** | **-** | - | - | - | - | - | - | - | - | - | - | - |
| **500** | **-** | **-** | - | - | - | - | - | - | - | - | - | - | - |
| **10** | **50** | **-** | - | - | - | - | - | - | - | - | - | - | - |
| **100** | **50** | **-** | - | - | - | - | - | - | - | - | - | - | - |
| **500** | **50** | **-** | - | - | - | - | - | - | - | - | - | - | - |

**Table S1.** Analysis of the growth of L2 and L4 complemented strains grown in DifcoNA (with or without 1% K_2_HPO_4_ addition) and different concentrations of lysozyme, vancomycin and teicoplanin. The concentration of the compounds is shown as µg per mL.
