# Supplementary material for: Genome sequencing analysis of Streptomyces coelicolor mutants that overcome the phosphate-depending vancomycin lethal effect
Source: BMC Genomics. 2018 Jun 14;19:457. doi: 10.1186/s12864-018-4838-z (PMC6001138; doi:10.1186/s12864-018-4838-z)
Supplement: Supplementary file 3 — Table S2. Analysis of the effect of coupling SCO1212 and SCO1213 to pVJ and pVJc on the growth of the next strains: SC100 (S. coelicolor M145-pVJ-1212; isolate 1), SC101 (S. coelicolor M145-pVJ-1212; isolate 2), SC102 (S. coelicolor M145-pVJc-1212; isolate 1), SC103 (S. coelicolor M145-pVJc-1212; isolate 2), SC104 (S. coelicolor M145-pVJ-1213; isolate 1), SC105 (S. coelicolor M145-pVJ-1213; isolate 2), SC106 (S. coelicolor M145-pVJc-1213; isolate 1), SC107 (S. coelicolor M145-pVJc-1213; isolate 2) in DifcoNA (with or without 1% K2HPO4 addition) and different concentrations of lysozyme, vancomycin and teicoplanin. The concentration of the compounds is shown as μg per mL. (DOCX 15 kb) [file 12864_2018_4838_MOESM3_ESM.docx]

|  | **DifcoNA (No Pi)** | | | | | | **DifcoNA (1%K_2_HPO_4_)** | | | | | |
| --- | --- | --- | --- | --- | --- | --- | --- | --- | --- | --- | --- | --- |
|  | **(-)** | **Tei5** | **Van50** | **Van50**  **Tei5** | **Lys100** | **Van50**  **Lys100** | **(-)** | **Tei5** | **Van50** | **Van50**  **Tei5** | **Lys100** | **Van50**  **Lys100** |
| **W1** | **++++** | - | **++++** | **++++** | **++++** | **++** | **++++** | - | - | - | - | - |
| **W2** | **++++** | - | **++++** | **++++** | **++++** | **++** | **++++** | - | - | - | - | - |
| **SC100** | **++++** | - | **++++** | **++++** | **++++** | **++** | **++++** | - | - | - | - | - |
| **SC101** | **++++** | - | **++++** | **++++** | **++++** | **++** | **++++** | - | - | - | - | - |
| **SC102** | **++++** | **++++** | **++++** | **++++** | **++** | **+** | **++++** | **++++** | **++++** | **++++** | - | - |
| **SC103** | **++++** | **++++** | **++++** | **++++** | **++** | **+** | **++++** | **++++** | **++++** | **++++** | - | - |
| **SC104** | **++++** | - | **++++** | **++++** | **++++** | **++** | **++++** | - | - | - | - | - |
| **SC105** | **++++** | - | **++++** | **++++** | **++++** | **++** | **++++** | - | - | - | - | - |
| **SC106** | **++++** | **++++** | **++++** | **++++** | **++** | **+** | **++++** | **++++** | **++++** | **++++** | - | - |
| **SC107** | **++++** | **++++** | **++++** | **++++** | **++** | **+** | **++++** | **++++** | **++++** | **++++** | - | - |

**Table S2.** Analysis of the effect of coupling SCO1212 and SCO1213 to pVJ and pVJc on the growth of the next strains: SC100 (*S. coelicolor* M145-pVJ-1212; isolate 1), SC101 (*S. coelicolor* M145-pVJ-1212; isolate 2), SC102 (*S. coelicolor* M145-pVJc-1212; isolate 1), SC103 (*S. coelicolor* M145-pVJc-1212; isolate 2), SC104 (*S. coelicolor* M145-pVJ-1213; isolate 1), SC105 (*S. coelicolor* M145-pVJ-1213; isolate 2), SC106 (*S. coelicolor* M145-pVJc-1213; isolate 1), SC107 (*S. coelicolor* M145-pVJc-1213; isolate 2) in DifcoNA (with or without 1% K_2_HPO_4_ addition) and different concentrations of lysozyme, vancomycin and teicoplanin. The concentration of the compounds is shown as µg per mL.
