# Supplementary material for: Genome sequencing analysis of Streptomyces coelicolor mutants that overcome the phosphate-depending vancomycin lethal effect
Source: BMC Genomics. 2018 Jun 14;19:457. doi: 10.1186/s12864-018-4838-z (PMC6001138; doi:10.1186/s12864-018-4838-z)
Supplement: Supplementary file 10 — Table S3. Primers used in this work. (DOCX 16 kb) [file 12864_2018_4838_MOESM10_ESM.docx]

**FSB29:** CTCTGGTACCTCACAAGCGTCAAGGACGGTCAGAGCTAC **(Dir for pVJ; KpnI)**

**FSB30:** GCGGAAGCTTGGAGCACCATATGTGACAGCCGTCTCCGAG **(Rev for pVJ; HindIII-NdeI)**

**FSB48:** GTTGCATATGATTGAACAAGATGGATTGCAC **(Dir *neo*; NdeI)**

**FSB49:** GTCGAAGCTTAGAGTCCCGCTCAGAAGAACTC **(Rev *neo*; HindIII)**

**FSB88**: GCCCGGCGGTACCTCGCTGTTCACCGGCATCGTCTTCATC **(Dir *rpsL_prom_*; KpnI)**

**FSB89:** GATCGTAGGCATATGTTCTCCGGTTTCTGTGTGCCGAATG **(Rev *rpsL_prom_*; NdeI)**

**FSB90:** CGACGACCGATCTGATGTGCTCAGTATC **(Dir for sequencing pVJ derivatives)**

**FSB101:** GCGGTGGAGTGCAATGTCGTGCAATAC **(Rev for sequencing pVJ derivatives)**

**FSB135:** GCTCCTACGACATAGTCATCCATCTGCAC **(Dir for sequencing chromosomal *vanSR* genes)**

**FSB136:** CCAGCAACAGCGCCGACACCGTGAGTAC **(Rev for sequencing chromosomal *vanSR* genes)**

**FSB184-L2:** GCCGCGATTCGGATCCGCGAATGGATCTCGATAG **(Dir SCO1212-13; BamHI)**

**FSB185-L2:** GGACACCGATATCCATAGCCCGAAATACCTTCTCAAC **(Rev SCO1212-13; EcoRV)**

**FSB184-L4:** GCCGCGATTCGGTACCGCGAATGGATCTCGATAG **(Dir SCO1212-13; KpnI)**

**FSB185-L4:** GGACACCAAGCTTCATAGCCCGAAATACCTTCTCAAC **(Rev SCO1212-13; HindIII)**

**FSB200:** CCAGACGGATATCAGTTGGTTGTCGCTCACGAAAATC **(Rev SCO1212; EcoRV)**

**FSB201:** CCAGACGAAGCTTAGTTGGTTGTCGCTCACGAAAATC **(Rev SCO1212; HindIII)**

**FSB202:** CGAGAACATATGGCAGGCAACTCGGACCCGCTCAC **(Dir SCO1212; NdeI)**

**FSB203:** AGCCCGAAGCTTCTTCTCAACGTGGCACGGGTCCGGAC **(Rev SCO1213; HindIII)**

**FSB204:** GGGGATTCATATGAGCGACAACCAACTGCGGATCGTCTG **(Dir SCO1213; NdeI)**

**Table S3.** Primers used in this work.
